# G Allele of the rs1801282 Polymorphism in PPARγ Gene Confers an Increased Risk of Obesity and Hypercholesterolemia, While T Allele of the rs3856806 Polymorphism Displays a Protective Role Against Dyslipidemia: A Systematic Review and Meta-Analysis

**DOI:** 10.3389/fendo.2022.919087

**Published:** 2022-06-29

**Authors:** Shujin Li, Chuan He, Haiyan Nie, Qianyin Pang, Ruixia Wang, Zhifu Zeng, Yongyan Song

**Affiliations:** ^1^ Central Laboratory, Clinical Medical College and Affiliated Hospital of Chengdu University, Chengdu, China; ^2^ Department of Cardiology, Clinical Medical College and Affiliated Hospital of Chengdu University, Chengdu, China; ^3^ Clinical Medical College of Chengdu University, Chengdu, China

**Keywords:** peroxisome proliferator-activated receptor gamma, polymorphism, rs1801282, rs3856806, obesity, dyslipidemia

## Abstract

**Background:**

The relationships between the rs1801282 and rs3856806 polymorphisms in nuclear receptor peroxisome proliferator-activated receptor gamma (PPARγ) gene and obesity indexes as well as serum lipid levels have been extensively investigated in various studies, but the results were inconsistent and even contradictory.

**Methods:**

PubMed, Google Scholar, Embase, Cochrane Library, Web of Science, Wanfang, CNKI and VIP databases were searched for eligible studies. The random-effTPDEects model was used, and standardized mean difference (SMD) with 95% confidence interval (CI) was calculated to estimate the differences in obesity indexes and serum lipid levels between the subjects with different genotypes in a dominant model. Heterogeneity among studies was assessed by Cochran’s x^2^-based Q-statistic test. Publication bias was identified by using Begg’s test.

**Results:**

One hundred and twenty studies (70,317 subjects) and 33 studies (18,353 subjects) were identified in the analyses for the rs1801282 and rs3856806 polymorphisms, respectively. The G allele carriers of the rs1801282 polymorphism had higher levels of body mass index (SMD = 0.08 kg/m^2^, 95% CI = 0.04 to 0.12 kg/m^2^, *p* < 0.001), waist circumference (SMD = 0.12 cm, 95% CI = 0.06 to 0.18 cm, *p* < 0.001) and total cholesterol (SMD = 0.07 mmol/L, 95% CI = 0.02 to 0.11 mmol/L, *p* < 0.01) than the CC homozygotes. The T allele carriers of the rs3856806 polymorphism had lower levels of low-density lipoprotein cholesterol (SMD = -0.09 mmol/L, 95% CI = -0.15 to -0.03 mmol/L, *p* < 0.01) and higher levels of high-density lipoprotein cholesterol (SMD = 0.06 mmol/L, 95% CI = 0.02 to 0.10 mmol/L, *p* < 0.01) than the CC homozygotes.

**Conclusions:**

The meta-analysis suggests that the G allele of the rs1801282 polymorphism confers an increased risk of obesity and hypercholesterolemia, while the T allele of the rs3856806 polymorphism displays a protective role against dyslipidemia, which can partly explain the associations between these polymorphisms and cardiovascular disease.

**Systematic Review Registration:**

https://www.crd.york.ac.uk/prospero/, identifier [CRD42022319347].

## Introduction

Peroxisome proliferator activated receptors (PPARs), belonging to the nuclear receptor superfamily, are ligand-inducible transcription factors (1). PPARs have three members in human beings: PPARα, PPARβ/δ and PPARγ. Of them, PPARγ is the most important one and plays an intricate role in various biological processes (2). Eight PPARγ isoforms (PPARγ1, PPARγ2, PPARγ3, etc.) have been identified in human beings according to NCBI’s reference sequence database (http://www.ncbi.nlm.nih.gov/). Upon activation by exogenous and endogenous lipid ligands, PPARγ binds to retinoid X receptor (RXR) to form a regulatory complex and is capable of stimulating adipogenesis (3), promoting adipocyte differentiation (4), and increasing insulin sensitivity (5). PPARγ is closely related to lipid disorders and obesity based on its fundamental role in lipid and glucose metabolism.

**Figure 1:**
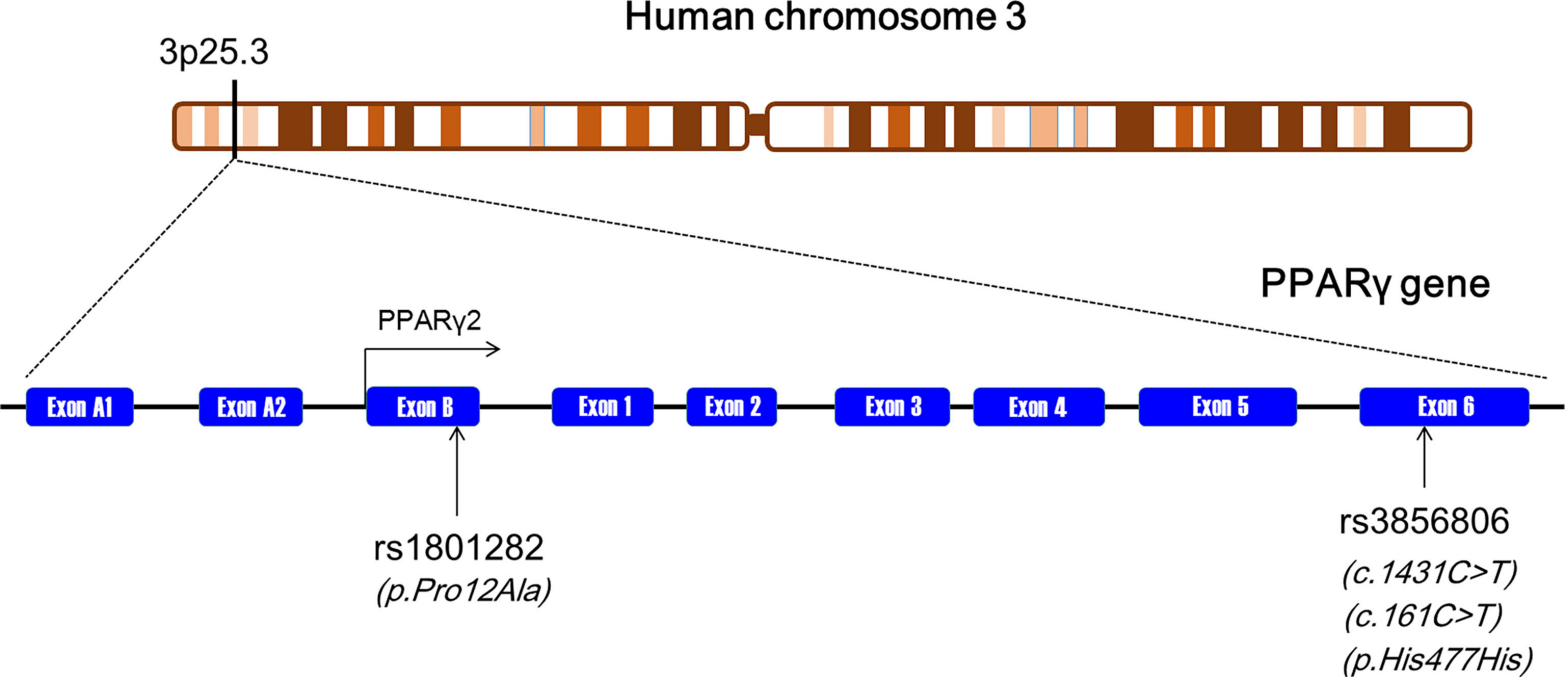
The genomic landscape of the rs1801282 and rs3856806 polymorphisms in PPARγ gene. PPARγ, peroxisome proliferator-activated receptor gamma.

Human PPARγ gene (namely *PPARG*) is located on chromosome 3p25.3 and consists of nine exons: exons A1, A2, B, and 1-6 (**Figure 1**) (2). According to NCBI’s RefSeq database, sixteen *PPARG* mRNA variants have been identified so far in human beings due to alternative splicing and differential promoter usage. PPARγ gene is highly polymorphic, and thousands of genetic variants have been recorded in NCBI’s dbSNP database. Among these variants, a missense variant (rs1801282, also known as p.Pro12Ala) located in exon B has been extensively explored with regard to its significant relationships with obesity indexes and serum lipid levels (**Figure 1**) (2). The rs1801282 polymorphism is formed by a single-nucleotide variance from cytosine (C) to guanine (G), resulting in a proline-to-alanine substitution in PPARγ2 polypeptide. Another genetic locus, the rs3856806 polymorphism (also known as p.His477His, c.161C>T or c.1431C>T), has also been investigated widely, although not as much as the rs1801282 polymorphism. The rs3856806 polymorphism is a synonymous variant and is located in exon 6 of *PPARG* (**Figure 1**). This genetic variation is formed by a single-nucleotide variance from C to thymine (T), but the corresponding amino acid residue in PPARγ2 polypeptide does not change after nucleotide substitution.

Scientific reports of the associations between the rs1801282 and rs3856806 polymorphisms and obesity indexes as well as serum lipid levels were inconsistent and even conflicting (2). Some studies indicated that the G allele of the rs1801282 polymorphism was associated with higher levels of body mass index (BMI) (6–17), waist circumference (WC) (17–20), waist-to-hip ratio (WHR) (14–18), total cholesterol (TC) (21–27), low-density lipoprotein cholesterol (LDL-C) (24–29) and triglycerides (TG) (30–38), and lower levels of high-density lipoprotein cholesterol (HDL-C) (38–41), whereas the research data from other laboratories did not support these findings and even yielded contradictory results (42–61). There were also significant inconsistencies amongst published data in the relationships between the rs3856806 polymorphism and obesity indexes as well as serum lipid levels in various populations (62–71).

Herein, a systematic review and meta-analysis was performed based on previous publications over the past two decades to determine the relationships between the rs1801282 and rs3856806 polymorphisms and obesity indexes as well as serum lipid levels. This work can provide an opportunity to unveil the interrelationships among PPARγ gene polymorphisms, metabolic disorders and cardiovascular disease.

## Methods

### Literature Search Strategy

The present meta-analysis was registered in PROSPERO (registration number CRD42022319347) and conducted according to the Preferred Reporting Items for Systematic Reviews and Meta-analysis (PRISMA) statement. PubMed, Google Scholar, Embase, Cochrane Library, Web of Science, Wanfang, CNKI and VIP databases were searched comprehensively from inception to December 2021. The keywords used for the literature searches were (“peroxisome proliferator-activated receptor gamma” or “PPARγ” or “PPARG”), (“polymorphism” or “mutation” or “variant” or “variance” or “rs1801282” or “rs3856806” or “Pro12Ala” or “1431C>T” or “161C>T” or “His477His”) and (“body mass index” or “waist circumference” or “waist-to-hip ratio” or “BMI” or “WC” or “WHR”) and (“lipid” or “total cholesterol” or “low-density lipoprotein cholesterol” or “high-density lipoprotein cholesterol” or “triglyceride” or “TC” or “LDL-C” or “HDL-C” or “TG”). The variables of this meta-analysis were limited to three obesity indexes including BMI, WC and WHR, and four serum lipid parameters including TC, LDL-C, HDL-C and TG. All articles that reported the associations of the rs1801282 and rs3856806 polymorphisms with obesity indexes and serum lipid levels were reviewed and screened.

### Inclusion and Exclusion Criteria

Inclusion criteria: 1) The sample size and genotype distribution were clearly provided; 2) At least one of the seven variables (i.e., BMI, WC, WHR, TG, TC, LDL-C, and HDL-C) was presented; 3) Data were displayed as mean ± standard deviation (SD) or mean ± standard error (SE). Exclusion criteria: 1) Animal studies; 2) Incomplete data; 3) Repeatedly published articles; 4) Case reports; 5) Conference abstracts.

### Data Extraction

Data were extracted independently by three reviewers. The data from each included study were as follows: first author’s name, year of publication, ethnicity, age, gender, health status, sample size, mean obesity indexes, mean lipid variables, and the SD or SE values by genotypes. SD values were calculated if SE values were given. Unit used for lipid variables was “mmol/L” in this meta-analysis, and datum conversion was conducted if data were presented as “mg/dL” or other units. All data were double-checked after extraction. Any disagreements were resolved by careful examination and group discussion.

### Meta-Analysis

The STATA software package (Version 10, StataCorp, USA) was used for the present meta-analysis. A dominant model was employed because most of the included studies reported results in a dominant way (i.e., CC vs [CG + GG] for the rs1801282 polymorphism; CC vs [CT + TT] for the rs3856806 polymorphism). If there were more than one subgroup in a study (e.g., the subgroups with different ethnicities or health conditions), each subgroup was treated as an independent comparison in the meta-analysis. The subgroup analyses were performed with at least 5 comparisons for the rs1801282 polymorphism, and 3 comparisons for the rs3856806 polymorphism to ensure adequate statistical power. Standardized mean difference (SMD) and 95% confidence interval (CI) were used to assess the differences in obesity indexes and serum lipid levels between the genotypes. The random-effects model was used in the meta-analysis for the reason that it provides a more conservative result than the fixed effects model. Heterogeneity among the included studies was assessed by Cochran’s x^2^-based Q-statistic test. Heterogeneity was considered statistically significant if *p* ≤ 0.05. Furthermore, subgroup analyses and Galbraith plots were applied to detect the potential sources of heterogeneity. Subgroup analyses were conducted according to ethnicities, health conditions, genders and ages of the subjects. The subgroups classified by ethnicity included European Caucasians, American Caucasians, Australian Caucasians, East Asians, South Asians, West Asians, South Americans, and Africans. The subgroups classified by health condition included coronary artery disease (CAD), type 2 diabetes mellitus (T2DM), metabolic syndrome (MetS), polycystic ovarian syndrome (PCOS), overweight/obesity, and general population/controls/healthy subjects; The subgroups classified by gender were males and females; The subgroups classified by age were adults (≥ 18 years) and children/adolescents (< 18 years). Publication bias was evaluated by using Begg’s test and visualized by Begg’s funnel plots, and *p* ≤ 0.05 the indicates the presence of a publication bias in the meta-analysis. The trim-and-fill method was used to adjust the results if a publication bias was present. All *p* values were two-tailed.

## Results

### Characteristics of the Enrolled Studies

**Figure 2:**
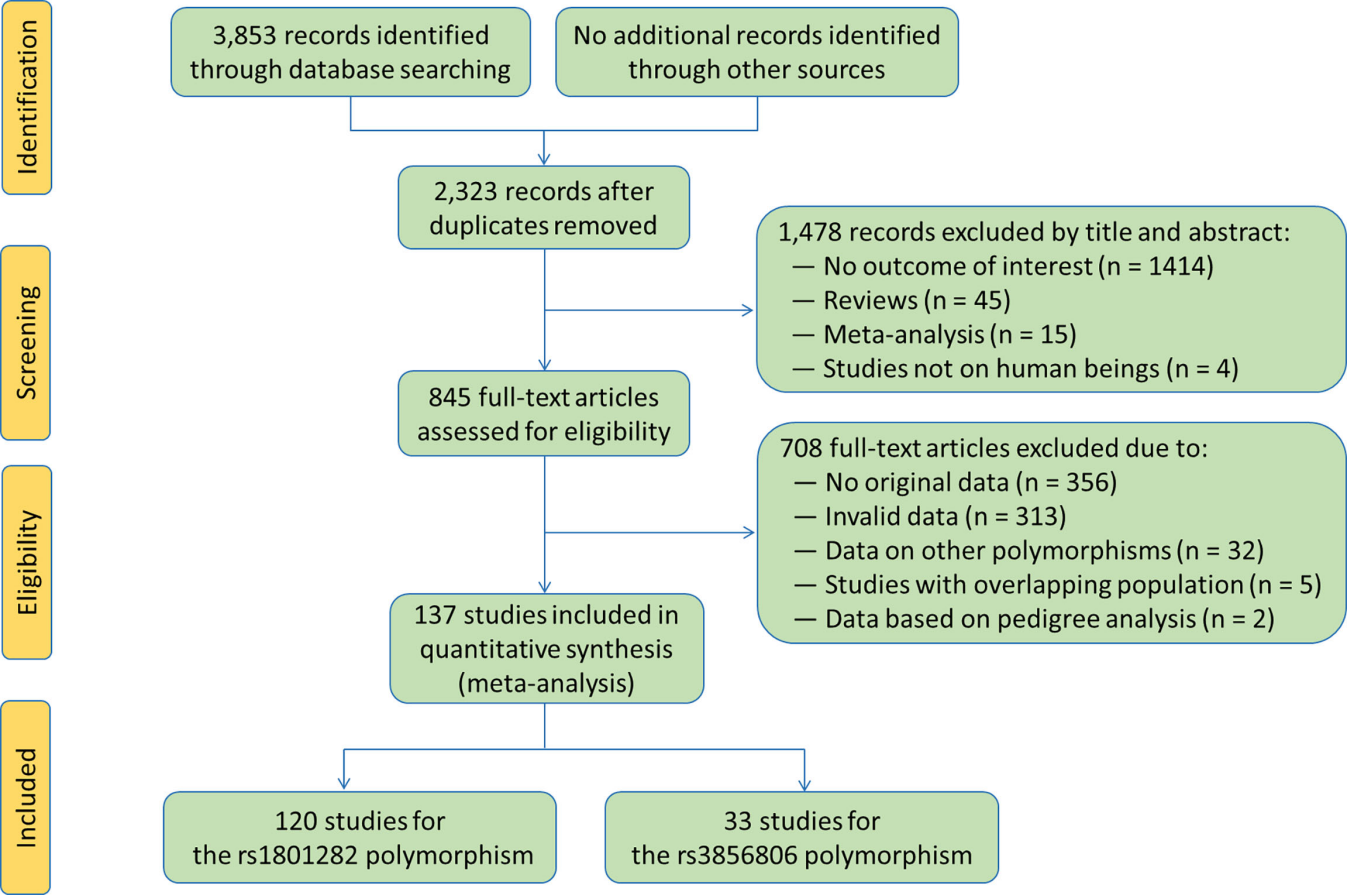
Flow chart of the literature selection in each stage.

The flow diagram of the literature search process is shown in **Figure 2**.** **A total of 137 studies (6–142) were identified and included in this meta-analysis. Characteristics of the included studies are presented in **Tables S1** and **S2**. The enrolled articles were published between 1998 and 2021, and written either in English (132 articles, 96.35%) or in Chinese (5 articles, 3.65%). Forty-eight studies, 5 studies, 2 studies, 44 studies, 9 studies, 8 studies, 7 studies, 7 studies and 7 studies involved European Caucasians, American Caucasians, Australian Caucasians, East Asians, South Asians, West Asians, South Americans, Africans and other ethnicities, respectively. Eleven studies, 29 studies, 4 studies, 10 studies, 23 studies and 77 studies involved CAD patients, T2DM patients, MetS patients, PCOS patients, overweight/obesity patients and general population/control subjects/healthy subjects, respectively. Six studies only involved males, 20 studies only involved females, and the rest studies involved both genders. One hundred and twenty-five studies involved adults, and the rest 12 studies involved children or adolescents. The subjects from 68 studies were divided into subgroups according to health conditions, genders or ethnicities, and each subgroup was considered as an independent comparison.

One hundred and twenty studies were enrolled in the meta-analysis for the rs1801282 polymorphism. Among them, 100 studies, 44 studies, 40 studies, 104 studies, 83 studies, 103 studies and 104 studies presented the data for BMI, WC, WHR, TC, LDL-C, HDL-C and TG, respectively (**Tables S3** and **S4**). Thirty-three studies were enrolled in the meta-analysis for the rs3856806 polymorphism, and 27 studies, 10 studies, 9 studies, 28 studies, 24 studies, 30 studies and 30 studies presented the data for BMI, WC, WHR, TC, LDL-C, HDL-C and TG, respectively (**Tables S5** and **S6**).

### Summary Statistics

One hundred and seventy-six comparisons (70,137 subjects) and 53 comparisons (18,353 subjects) were distinguished for the rs1801282 and rs3856806 polymorphisms, respectively. One hundred and fifty comparisons, 168 comparisons, 54 comparisons, 142 comparisons, 117 comparisons, 146 comparisons and 151 comparisons were enrolled to compare the differences in BMI, WC, WHR, TC, LDL-C, HDL-C and TG levels for the rs1801282 polymorphism, respectively **(** **Tables S3** and **S4** **)**. Forty-five comparisons, 16 comparisons, 14 comparisons, 46 comparisons, 41 comparisons, 49 comparisons and 50 comparisons were enrolled to compare the differences in BMI, WC, WHR, TC, LDL-C, HDL-C and TG levels, respectively, for the rs3856806 polymorphism **(** **Tables S5** and **S6** **)**. For the rs1801282 polymorphism, 83.32% of the subjects had CC genotype (58,438 subjects), and 16.94% of the subjects had CG or GG genotype (11,879 subjects). Regarding the rs3856806 polymorphism, 66.06% of the subjects had CC genotype (12,124 subjects), and 33.94% of the subjects had CT or TT genotype (6,229 subjects).

### Associations of the *PPARG* rs1801282 Polymorphism With Obesity Indexes and Serum Lipid Levels

The associations between the rs1801282 polymorphism and obesity indexes are shown in **Table 1**. The pooled analyses in the whole population showed that the G allele carriers had significantly higher levels of BMI (SMD = 0.08 kg/m^2^, 95% CI = 0.04 to 0.12 kg/m^2^, *p* < 0.001) and WC (SMD = 0.12 cm, 95% CI = 0.06 to 0.18 cm, *p* < 0.001) than the CC homozygotes. The G allele carriers also had marginally insignificantly higher levels of WHR (SMD = 0.08, 95% CI = -0.01 to 0.17, *p* = 0.08) than the CC homozygotes. The associations between the rs1801282 polymorphism and serum lipid levels are shown in **Table 2**. The pooled analyses in the whole population showed that the G allele carriers had significantly higher levels of TC (SMD = 0.07 mmol/L, 95% CI = 0.02 to 0.11 mmol/L, *p* < 0.01) than the CC homozygotes. There were no significant differences in LDL-C, HDL-C or TG levels between the subjects with different genotypes of the rs1801282 polymorphism (**Table 2**).

**Table 1:** Meta-analysis between the rs1801282 polymorphism in *PPARG* and obesity indexes.

Groups or subgroups	Comparisons (Subjects)	SMD (95% CI)	*P*_Heterogeneity_	*P*_SMD_
BMI				
All subjects	143 (57,461)	0.08 (0.04, 0.12)	< 0.001	< 0.001
East Asians	38 (14,512)	0.12 (0.03, 0.20)	0.01	0.01
South Asians	11 (6,254)	0.23 (0.02, 0.45)	< 0.001	0.03
West Asians	12 (2,171)	0.15 (-0.07, 0.36)	0.001	0.21
European Caucasians	54 (19,913)	0.01 (-0.04, 0.06)	0.01	0.65
American Caucasians	5 (4,118)	0.07 (-0.003, 0.15)	0.53	0.06
South Americans	8 (1,692)	-0.02 (-0.20, 0.15)	0.22	0.78
Africans	9 (2,370)	0.35 (-0.06, 0.75)	< 0.001	0.09
CAD patients	7 (1,395)	0.40 (-0.06, 0.86)	< 0.001	0.09
T2DM patients	27 (8,506)	0.13 (0.02, 0.23)	< 0.001	0.02
Overweight/obesity patients	19 (4,600)	0.16 (0.01, 0.31)	< 0.001	0.04
MetS patients	5 (648)	0.09 (-0.23, 0.41)	0.16	0.57
PCOS patients	9 (1,212)	-0.14 (-0.31, 0.03)	0.35	0.10
General population/control subjects/healthy subjects	70 (37,286)	0.05 (0.01, 0.09)	< 0.001	0.02
Adults	131 (51,030)	0.09 (0.05, 0.13)	< 0.001	< 0.001
Children/adolescents	12 (6,431)	-0.02 (-0.09, 0.04)	0.77	0.50
Males	20 (7,376)	0.08 (0.004, 0.16)	0.10	0.04
Females	38 (11,576)	0.03 (-0.04, 0.10)	0.01	0.47
WC				
All subjects	71 (31,963)	0.12 (0.06, 0.18)	< 0.001	< 0.001
East Asians	14 (4,409)	0.14 (0.02, 0.25)	0.17	0.03
South Asians	6 (1,891)	0.14 (-0.18, 0.46)	< 0.001	0.39
West Asians	6 (812)	-0.004 (-0.29, 0.28)	0.001	0.21
European Caucasians	28 (14,483)	0.04 (-0.01, 0.09)	0.36	0.09
South Americans	6 (726)	0.03 (-0.17, 0.23)	0.49	0.77
Africans	6 (1,741)	1.02 (0.09, 1.94)	< 0.001	0.03
T2DM patients	7 (1,114)	0.33 (0.14, 0.52)	0.19	0.001
Overweight/obesity patients	11 (3,649)	0.13 (-0.02, 0.28)	0.01	0.09
MetS patients	5 (648)	-0.01 (-0.25, 0.24)	0.56	0.96
General population/control subjects/healthy subjects	40 (21,337)	0.06 (0.01, 0.11)	0.02	0.02
Adults	63 (26,705)	0.14 (0.07, 0.21)	< 0.001	< 0.001
Children/adolescents	8 (5,258)	0.02 (-0.07, 0.11)	0.28	0.63
Males	11 (4,309)	0.05 (-0.05, 0.15)	0.22	0.30
Females	21 (5,685)	0.24 (0.04, 0.44)	< 0.001	0.02
WHR				
All subjects	51 (18,050)	0.08 (-0.01, 0.17)	< 0.001	0.08
East Asians	15 (5,250)	0.23 (0.09, 0.37)	0.02	< 0.01
South Asians	5 (1,717)	0.06 (-0.26, 0.38)	< 0.01	0.72
European Caucasians	19 (6,490)	-0.06 (-0.18, 0.07)	< 0.001	0.36
T2DM patients	7 (2,424)	0.25 (0.08, 0.43)	0.06	< 0.01
Overweight/obesity patients	6 (2,533)	0.12 (-0.10, 0.35)	0.03	0.28
PCOS patients	6 (715)	0.35 (-0.41, 1.10)	< 0.001	0.37
General population/control subjects/healthy subjects	26 (10,992)	-0.05 (-0.15, 0.05)	< 0.001	0.31
Adults	46 (16,182)	0.10 (0.001, 0.19)	< 0.001	0.05
Children/adolescents	5 (1,868)	-0.02 (-0.13, 0.08)	0.78	0.68
Males	5 (1,348)	0.03 (-0.10, 0.15)	0.81	0.68
Females	20 (5,592)	0.07 (-0.15, 0.29)	< 0.001	0.54

PPARG, peroxisome proliferator-activated receptor gamma gene; SMD, standardized mean difference; CI, confidence interval; BMI, body mass index; CAD, coronary artery disease; T2DM, type 2 diabetes mellitus; MetS, metabolic syndrome; PCOS, polycystic ovarian syndrome; WC, waist circumference; WHR, waist-to-hip ratio.

**Table 2:** Meta-analysis between the rs1801282 polymorphism in *PPARG* and serum lipid levels.

Groups or subgroups	Comparisons (Subjects)	SMD (95% CI)	*P*_Heterogeneity_	*P*_SMD_
TC				
All subjects	141 (48,494)	0.07 (0.02, 0.11)	< 0.001	< 0.01
East Asians	39 (13,645)	0.11 (0.02, 0.20)	< 0.001	0.02
South Asians	10 (7,328)	0.07 (-0.01, 0.14)	0.41	0.07
West Asians	11 (1,777)	0.19 (0.02, 0.36)	0.03	0.03
European Caucasians	48 (16,667)	0.01 (-0.04, 0.06)	0.04	0.67
South Americans	10 (1,929)	-0.06 (-0.25, 0.13)	0.05	0.52
Africans	9 (1,442)	0.68 (-0.03, 1.39)	< 0.001	0.06
CAD patients	8 (1,658)	0.48 (0.15, 0.82)	< 0.001	0.01
T2DM patients	23 (7,657)	0.11 (0.03, 0.20)	0.07	0.01
Overweight/obesity patients	22 (5,333)	0.07 (-0.02, 0.16)	0.19	0.11
MetS patients	5 (648)	0.26 (0.02, 0.51)	0.54	0.04
PCOS patients	9 (1,314)	0.58 (-0.13, 1.28)	< 0.001	0.11
General population/control subjects/healthy subjects	61 (27,499)	0.01 (-0.04, 0.06)	< 0.001	0.73
Adults	124 (43,583)	0.08 (0.03, 0.13)	< 0.001	< 0.01
Children/adolescents	14 (4,911)	-0.03 (-0.11, 0.05)	0.36	0.43
Males	15 (5,218)	0.01 (-0.06, 0.08)	0.42	0.79
Females	33 (8,869)	0.08 (-0.07, 0.23)	< 0.001	0.31
LDL-C				
All subjects	117 (37,849)	0.02 (-0.02, 0.07)	< 0.001	0.28
East Asians	28 (8,004)	0.07 (-0.04, 0.17)	< 0.01	0.21
South Asians	7 (6,029)	0.02 (-0.06, 0.09)	0.83	0.65
West Asians	9 (1,465)	0.14 (-0.02, 0.31)	0.14	0.09
European Caucasians	45 (14,672)	-0.03 (-0.08, 0.03)	0.02	0.36
South Americans	7 (1,102)	-0.20 (-0.41, 0.02)	0.15	0.07
Africans	8 (1,200)	0.46 (-0.01, 0.94)	< 0.001	0.06
CAD patients	7 (1,440)	0.39 (-0.08, 0.87)	< 0.001	0.11
T2DM patients	19 (4,406)	0.01 (-0.10, 0.13)	0.02	0.80
Overweight/obesity patients	20 (4,831)	0.05 (-0.03, 0.12)	0.80	0.23
MetS patients	5 (882)	0.26 (0.02, 0.49)	0.87	0.03
PCOS patients	8 (1,130)	0.33 (-0.15, 0.81)	< 0.001	0.18
General population/control subjects/healthy subjects	48 (21,623)	0.004 (-0.04, 0.04)	0.14	0.86
Adults	100 (32,938)	0.03 (-0.02, 0.08)	< 0.001	0.22
Children/adolescents	14 (4,911)	-0.03 (-0.10, 0.04)	0.69	0.40
Males	15 (5,142)	-0.04 (-0.16, 0.07)	0.01	0.45
Females	30 (7,983)	0.08 (-0.03, 0.19)	< 0.001	0.14
HDL-C				
All subjects	144 (52,515)	0.004 (-0.04, 0.04)	< 0.001	0.85
East Asians	34 (9,600)	-0.02 (-0.13, 0.08)	< 0.001	0.64
South Asians	11 (7,579)	-0.09 (-0.25, 0.08)	< 0.001	0.31
West Asians	11 (1,777)	-0.09 (-0.28, 0.11)	< 0.01	0.38
European Caucasians	57 (22,402)	0.03 (-0.03, 0.08)	< 0.001	0.36
American Caucasians	5 (3,635)	0.04 (-0.04, 0.12)	0.55	0.38
South Americans	10 (1,929)	0.06 (-0.06, 0.19)	0.73	0.33
Africans	7 (2,196)	0.09 (-0.08, 0.26)	0.37	0.31
CAD patients	7 (1,440)	-0.10 (-0.42, 0.23)	< 0.001	0.56
T2DM patients	21 (5,403)	0.08 (-0.02, 0.17)	0.08	0.11
Overweight/obesity patients	24 (6,204)	-0.11 (-0.24, 0.02)	< 0.001	0.11
MetS patients	5 (648)	0.04 (-0.23, 0.30)	0.34	0.79
PCOS patients	8 (1,214)	0.44 (-0.03, 0.90)	< 0.001	0.07
General population/control subjects/healthy subjects	61 (28,523)	0.01 (-0.02, 0.05)	0.40	0.40
Adults	128 (48,016)	0.01 (-0.04, 0.05)	< 0.001	0.74
Children/adolescents	13 (4,499)	-0.001 (-0.08, 0.08)	0.41	0.99
Males	18 (5,765)	-0.04 (-0.11, 0.03)	0.54	0.28
Females	34 (9,411)	0.09 (-0.01, 0.18)	< 0.001	0.07
TG				
All subjects	146 (53,204)	0.04 (-0.02, 0.11)	< 0.001	0.18
East Asians	36 (11,861)	0.11 (0.01, 0.21)	< 0.001	0.03
South Asians	11 (7,598)	0.004 (-0.16, 0.16)	< 0.001	0.96
West Asians	11 (1,777)	0.33 (0.09, 0.57)	< 0.001	0.01
European Caucasians	52 (20,331)	-0.04 (-0.16, 0.09)	< 0.001	0.58
American Caucasians	5 (3,635)	-0.02 (-0.09, 0.07)	0.94	0.72
South Americans	9 (1,208)	-0.10 (-0.30, 0.11)	0.10	0.37
Africans	12 (3,141)	0.14 (-0.13, 0.40)	< 0.001	0.31
CAD patients	9 (1,897)	0.09 (-0.07, 0.25)	0.17	0.25
T2DM patients	22 (6,261)	0.13 (-0.39, 0.12)	< 0.001	0.30
Overweight/obesity patients	23 (6,031)	0.05 (-0.08, 0.17)	< 0.001	0.47
PCOS patients	8 (1,130)	0.32 (-0.15, 0.78)	< 0.001	0.18
General population/control subjects/healthy subjects	64 (28,505)	0.07 (-0.02, 0.16)	< 0.001	0.14
Adults	129 (48,449)	0.05 (-0.02, 0.12)	< 0.001	0.15
Children/adolescents	13 (4,499)	-0.01 (-0.10, 0.07)	0.32	0.79
Males	17 (5,910)	0.03 (-0.05, 0.10)	0.33	0.50
Females	32 (7,502)	0.07 (-0.05, 0.18)	< 0.001	0.29

PPARG, peroxisome proliferator-activated receptor gamma gene; SMD, standardized mean difference; CI, confidence interval; TC, total cholesterol; CAD, coronary artery disease; T2DM, type 2 diabetes mellitus; MetS, metabolic syndrome; PCOS, polycystic ovarian syndrome; LDL-C, low-density lipoprotein cholesterol; HDL-C, high-density lipoprotein cholesterol; TG, triglyceride.

Subgroup analyses were conducted according to ethnicities, health conditions, ages and genders of the subjects. In East Asians, the G allele carriers had higher levels of BMI (SMD = 0.12 kg/m^2^, 95% CI = 0.03 to 0.20 kg/m^2^, *p* = 0.01), WC (SMD = 0.14 cm, 95% CI = 0.02 to 0.25 cm, *p* = 0.03), WHR (SMD = 0.23, 95% CI = 0.09 to 0.37, *p* < 0.01), TC (SMD = 0.11 mmol/L, 95% CI = 0.02 to 0.20 mmol/L, *p* = 0.02) and TG (SMD = 0.11 mmol/L, 95% CI = 0.01 to 0.21 mmol/L, *p* = 0.03) than the CC homozygotes. In West Asians, the G allele carriers had higher levels of TC (SMD = 0.19 mmol/L, 95% CI = 0.02 to 0.36 mmol/L, *p* = 0.03) and TG (SMD = 0.33 mmol/L, 95% CI = 0.09 to 0.57 mmol/L, *p* = 0.01) than the CC homozygotes. The G allele carriers had higher levels of BMI (SMD = 0.23 kg/m^2^, 95% CI = 0.02 to 0.45 kg/m^2^, *p* = 0.03) and WC (SMD = 1.02 cm, 95% CI = 0.09 to 1.94 cm, *p* < 0.03) than non-carriers in South Asians and Africans, respectively. Notably, no significant associations between the rs1801282 polymorphism and obesity indexes or serum lipid levels were detected in European Caucasians and American Caucasians. In patients with T2DM, the G allele carriers had higher levels of BMI (SMD = 0.13 kg/m^2^, 95% CI = 0.02 to 0.23 kg/m^2^, *p* = 0.02), WC (SMD = 0.33 cm, 95% CI = 0.14 to 0.52 cm, *p* = 0.001), WHR (SMD = 0.25, 95% CI = 0.08 to 0.43, *p* < 0.01) and TC (SMD = 0.11 mmol/L, 95% CI = 0.03 to 0.20 mmol/L, *p* = 0.01) than the CC homozygotes. In patients with MetS, the G allele carriers had higher levels of TC (SMD = 0.26 mmol/L, 95% CI = 0.02 to 0.51 mmol/L, *p* = 0.04) and LDL-C (SMD = 0.26 mmol/L, 95% CI = 0.02 to 0.49 mmol/L, *p* = 0.03) than the CC homozygotes. The G allele carriers had higher levels of BMI (SMD = 0.16 kg/m^2^, 95% CI = 0.01 to 0.31 kg/m^2^, *p* = 0.04) and TC (SMD = 0.48 mmol/L, 95% CI = 0.15 to 0.82 mmol/L, *p* = 0.01) than non-carriers in overweight/obesity patients and CAD patients, respectively. In general population/control subjects/healthy subjects, the G allele carriers had higher levels of BMI (SMD = 0.05 kg/m^2^, 95% CI = 0.01 to 0.09 kg/m^2^, *p* = 0.02) and WC (SMD = 0.06 cm, 95% CI = 0.01 to 0.11 cm, *p* = 0.02) than the CC homozygotes.

Significant interactions between the rs1801282 polymorphism and age as well as gender on obesity indexes or serum lipid levels have been detected. The G allele carriers had higher levels of BMI (SMD = 0.09 kg/m^2^, 95% CI = 0.05 to 0.13 kg/m^2^, *p* < 0.001), WC (SMD = 0.14 cm, 95% CI = 0.07 to 0.21 cm, *p* < 0.001), WHR (SMD = 0.10, 95% CI = 0.001 to 0.19, *p* = 0.05) and TC (SMD = 0.08 mmol/L, 95% CI = 0.03 to 0.13 mmol/L, *p* < 0.01) than the CC homozygotes in adults, but not in children and adolescents. Higher levels of BMI (SMD = 0.08 kg/m^2^, 95% CI = 0.004 to 0.16 kg/m^2^, *p* = 0.04) in the G allele carriers than in the CC homozygotes were observed only in males, and higher levels of WC (SMD = 0.24 cm, 95% CI = 0.04 to 0.44 cm, *p* = 0.02) in the G allele carriers than in the CC homozygotes were present only in females.

### Associations of the *PPARG* rs3856806 Polymorphism With Obesity Indexes and Serum Lipid Levels

As shown in **Table 3**, no significant associations between the rs3856806 polymorphism and obesity indexes were found in the pooled analyses in the whole population or in the subgroups according to ethnicities, health conditions or genders of the subjects. The associations between the rs3856806 polymorphism and serum lipid levels are shown in **Table 4**. The pooled analyses in the whole population showed that the T allele carriers had lower levels of LDL-C (SMD = -0.09 mmol/L, 95% CI = -0.15 to -0.03 mmol/L, *p* < 0.01) and higher levels of HDL-C (SMD = 0.06 mmol/L, 95% CI = 0.02 to 0.10 mmol/L, *p* < 0.01) than the CC homozygotes. There were no significant differences in TC or TG levels between the subjects with different genotypes of the rs3856806 polymorphism (**Table 4**). Subgroup analyses were conducted according to ethnicities, health conditions and genders of the subjects. Reduced levels of TC (SMD = -0.22 mmol/L, 95% CI = -0.35 to -0.08 mmol/L, *p* < 0.01), LDL-C (SMD = -0.26 mmol/L, 95% CI = -0.49 to -0.03 mmol/L, *p* = 0.03) and TG (SMD = -0.14 mmol/L, 95% CI = -0.26 to -0.02 mmol/L, *p* = 0.02) in the T allele carriers than in the CC homozygotes were detected in Australian Caucasians, but not in European Caucasians, American Caucasians or other ethnicities. The T allele carriers had higher levels of HDL-C (SMD = 0.15 mmol/L, 95% CI = 0.04 to 0.27 mmol/L, *p* = 0.01) than the CC homozygotes in patients with CAD, but not in patients with other clinical symptoms or in general population/control subjects/healthy subjects.

**Table 3:** Meta-analysis between the rs3856806 polymorphism in *PPARG* and obesity indexes.

Groups or subgroups	Comparisons (Subjects)	SMD (95% CI)	*P*_Heterogeneity_	*P*_SMD_
BMI				
All subjects	45 (16,600)	-0.01 (-0.07, 0.05)	< 0.001	0.78
East Asians	20 (5,707)	0.04 (-0.08, 0.15)	< 0.001	0.56
South Asians	7 (5,333)	-0.05 (-0.17, 0.06)	0.03	0.35
West Asians	3 (307)	-0.16 (-0.39, 0.07)	0.36	0.18
European Caucasians	7 (2,772)	0.05 (-0.11, 0.21)	0.01	0.54
Australian Caucasians	3 (1,294)	-0.03 (-0.14, 0.09)	0.57	0.68
CAD patients	6 (1,863)	-0.04 (-0.24, 0.17)	< 0.01	0.73
T2DM patients	7 (1,375)	-0.08 (-0.24, 0.09)	0.10	0.36
Overweight/obesity patients	5 (556)	-0.11 (-0.85, 0.63)	< 0.001	0.77
General population/control subjects/healthy subjects	16 (9,450)	0.03 (-0.06, 0.11)	0.001	0.54
Males	6 (1,381)	0.39 (-0.04, 0.83)	< 0.001	0.08
Females	5 (1,087)	-0.16 (-0.45, 0.12)	< 0.001	0.27
WC				
All subjects	16 (5,787)	0.003 (-0.06, 0.07)	0.32	0.93
East Asians	4 (1,644)	0.01 (-0.14, 0.17)	0.09	0.87
South Asians	3 (1,021)	-0.11 (-0.37, 0.14)	0.15	0.39
Australian Caucasians	3 (1,294)	0.04 (-0.08, 0.16)	0.38	0.53
European Caucasians	3 (837)	0.01 (-0.20, 0.22)	0.26	0.92
General population/control subjects/healthy subjects	8 (3,352)	-0.01 (-0.09, 0.07)	0.79	0.77
Males	3 (1,003)	0.05 (-0.18, 0.28)	0.09	0.68
Females	5 (1,333)	0.02 (-0.12, 0.17)	0.24	0.77
WHR				
All subjects	14 (5,198)	-0.02 (-0.11, 0.06)	0.09	0.58
East Asians	3 (984)	0.00 (-0.13, 0.13)	1.00	1.00
Australian Caucasians	3 (1,294)	-0.11 (-0.37, 0.16)	0.01	0.42
European Caucasians	3 (1,090)	-0.03 (-0.13, 0.18)	0.31	0.73
General population/control subjects/healthy subjects	8 (3,485)	-0.02 (-0.12, 0.08)	0.17	0.72

PPARG, peroxisome proliferator-activated receptor gamma gene; SMD, standardized mean difference; CI, confidence interval; BMI, body mass index; CAD, coronary artery disease; T2DM, type 2 diabetes mellitus; WC, waist circumference; WHR, waist-to-hip ratio.

**Table 4:** Meta-analysis between the rs3856806 polymorphism in *PPARG* and serum lipid levels.

Groups or subgroups	Comparisons (Subjects)	SMD (95% CI)	*P*_Heterogeneity_	*P*_SMD_
TC				
All subjects	46 (16,716)	-0.04 (-0.09, 0.02)	< 0.001	0.18
East Asians	24 (6,999)	-0.03 (-0.10, 0.05)	< 0.01	0.52
South Asians	6 (5,059)	0.03 (-0.03, 0.09)	0.44	0.34
West Asians	4 (590)	-0.03 (-0.32, 0.27)	0.04	0.87
Australian Caucasians	3 (1,294)	-0.22 (-0.35, -0.08)	0.30	< 0.01
European Caucasians	4 (1,587)	0.03 (-0.14, 0.19)	0.16	0.76
CAD patients	8 (2,587)	-0.01 (-0.13, 0.12)	0.05	0.90
T2DM patients	7 (1,375)	-0.02 (-0.19, 0.16)	0.07	0.85
Overweight/obesity patients	5 (556)	0.06 (-0.19, 0.30)	0.28	0.66
General population/control subjects/healthy subjects	15 (9,143)	0.02 (-0.06, 0.09)	0.01	0.60
Males	3 (568)	-0.001 (-0.28, 0.27)	0.95	1.00
Females	5 (1,018)	-0.10 (-0.23, 0.03)	< 0.001	0.14
LDL-C				
All subjects	41 (14,279)	-0.09 (-0.15, -0.03)	< 0.001	< 0.01
East Asians	22 (6,067)	-0.08 (-0.16, 0.01)	< 0.01	0.08
South Asians	5 (4,373)	0.01 (-0.05, 0.08)	0.96	0.67
West Asians	4 (590)	-0.05 (-0.35, 0.24)	0.04	0.72
Australian Caucasians	3 (1,294)	-0.26 (-0.49, -0.03)	0.04	0.03
CAD patients	8 (2,182)	0.01 (-0.12, 0.13)	0.06	0.93
T2DM patients	6 (1,235)	-0.06 (-0.23, 0.11)	0.13	0.51
Overweight/obesity patients	4 (440)	0.03 (-0.23, 0.28)	0.57	0.83
General population/control subjects/healthy subjects	12 (6,962)	-0.09 (-0.18, 0.01)	0.02	0.04
Males	3 (568)	-0.02 (-0.64, 0.60)	0.02	0.95
Females	5 (1,018)	-0.11 (-0.24, 0.02)	0.96	0.10
HDL-C				
All subjects	49 (17,161)	0.06 (0.02, 0.10)	0.10	< 0.01
East Asians	25 (6,806)	0.06 (-0.02, 0.13)	0.02	0.12
South Asians	7 (4,911)	0.01 (-0.04, 0.07)	0.52	0.62
West Asians	4 (590)	0.02 (-0.18, 0.21)	0.28	0.86
European Caucasians	5 (1,953)	0.06 (-0.05, 0.17)	0.67	0.28
Australian Caucasians	3 (1,294)	0.11 (-0.01, 0.23)	0.91	0.08
CAD patients	8 (2,182)	0.15 (0.04, 0.27)	0.10	0.01
T2DM patients	7 (1,375)	0.12 (-0.04, 0.29)	0.09	0.15
Overweight/obesity patients	4 (440)	-0.12 (-0.38, 0.13)	0.86	0.35
General population/control subjects/healthy subjects	17 (9,105)	0.07 (0.02, 0.12)	0.33	< 0.01
Males	5 (1,087)	-0.05 (-0.21, 0.11)	0.30	0.56
Females	7 (1,664)	-0.03 (-0.13, 0.08)	0.90	0.65
TG				
All subjects	50 (17,357)	-0.06 (-0.12, 0.01)	< 0.001	0.10
East Asians	25 (6,806)	-0.06 (-0.18, 0.06)	< 0.001	0.34
South Asians	7 (5,327)	-0.02 (-0.08, 0.04)	0.76	0.49
West Asians	4 (590)	0.09 (-0.23, 0.41)	0.03	0.59
Australian Caucasians	3 (1,294)	-0.14 (-0.26, -0.02)	0.82	0.02
European Caucasians	6 (2,153)	0.02 (-0.08, 0.13)	0.67	0.68
CAD patients	8 (2,587)	-0.15 (-0.34, 0.04)	< 0.001	0.12
T2DM patients	7 (1,375)	-0.14 (-0.52, 0.24)	< 0.001	0.48
Overweight/obesity patients	5 (556)	-0.17 (-0.42, 0.08)	0.28	0.19
General population/control subjects/healthy subjects	17 (9,185)	0.06 (-0.03, 0.15)	< 0.001	0.21
Males	5 (1,087)	-0.01 (-0.14, 0.13)	0.48	0.92
Females	7 (1,664)	-0.03 (-0.15, 0.10)	0.22	0.69

PPARG, peroxisome proliferator-activated receptor gamma gene; SMD, standardized mean difference; CI, confidence interval; TC, total cholesterol; CAD, coronary artery disease; T2DM, type 2 diabetes mellitus; LDL-C, low-density lipoprotein cholesterol; HDL-C, high-density lipoprotein cholesterol; TG, triglycerides.

### Heterogeneity Analysis

Galbraith plots were employed to analyze the heterogeneity in the present meta-analysis. For the rs1801282 polymorphism, there was significant heterogeneity in the pooled analyses in the whole population for all three obesity indexes **(** **Table 1** **)** and four lipid variables **(** **Table 2** **)**. Twelve comparisons, 7 comparisons, 6 comparisons, 6 comparisons, 6 comparisons, 11 comparisons and 18 comparisons were identified as the main contributors to the heterogeneity for the analyses of BMI, WC, WHR, TC, LDL-C, HDL-C and TG, respectively **(** **Table S7** **)**. The heterogeneity was significantly decreased or removed after exclusion of the outlier comparisons, while the results of the pooled analyses in the whole population did not change significantly (BMI: SMD = 0.04 kg/m^2^, 95% CI = 0.02 to 0.07 kg/m^2^, *P*_SMD_ < 0.01, *P*_Heterogeneity_ = 0.30; WC: SMD = 0.06 cm, 95% CI = 0.02 to 0.09 cm, *P*_SMD_ < 0.01, *P*_Heterogeneity_ = 0.22; WHR: SMD = 0.03 kg/m^2^, 95% CI = -0.02 to 0.08 kg/m^2^, *P*_SMD_ = 0.20, *P*_Heterogeneity_ = 0.15; TC: SMD = 0.02 mmol/L, 95% CI = 0.01 to 0.04 mmol/L, *P*_SMD_ = 0.02, *P*_Heterogeneity_ = 0.44; LDL-C: SMD = 0.01 mmol/L, 95% CI = -0.02 to 0.03 mmol/L, *P*_SMD_ = 0.66, *P*_Heterogeneity_ = 0.64; HDL-C: SMD = 0.01 mmol/L, 95% CI = -0.01 to 0.04 mmol/L, *P*_SMD_ = 0.36, *P*_Heterogeneity_ = 0.84; TG: SMD = -0.02 mmol/L, 95% CI = -0.05 to 0.003 mmol/L, *P*_SMD_ = 0.09, *P*_Heterogeneity_ = 0.55).

Regarding the rs3856806 polymorphism, there was significant heterogeneity in the pooled analyses in the whole population for BMI, TC, LDL-C and TG (**Tables 3** and **4**). Five comparisons, 6 comparisons, 4 comparisons and 7 comparisons were identified as the main contributors to the heterogeneity in the association analyses in the whole population between the rs3856806 polymorphism and BMI, TC, LDL-C and TG, respectively (**Table S8**). The heterogeneity was significantly decreased or removed after exclusion of the outlier studies, and the pooled results in the whole population did not change significantly for BMI (SMD = -0.001 kg/m^2^, 95% CI = -0.04 to 0.04 kg/m^2^, *P*_SMD_ = 0.96, *P*_Heterogeneity_ = 0.18), TC (SMD = -0.02 mmol/L, 95% CI = -0.06 to 0.02 mmol/L, *P*_SMD_ = 0.26, *P*_Heterogeneity_ = 0.30), and LDL-C (SMD = -0.05 mmol/L, 95% CI = -0.09 to -0.01 mmol/L, *P*_SMD_ < 0.01, *P*_Heterogeneity_ = 0.46). However, the pooled results for TG became significant after exclusion of the outlier studies (SMD = -0.04 mmol/L, 95% CI = -0.08 to -0.003 mmol/L, *P*_SMD_ = 0.04, *P*_Heterogeneity_ = 0.23).

### Publication Bias

Begg’s test was conducted to identify the publication bias in the present meta-analysis. No publication bias was found in the association analyses between the rs1801282 polymorphism and BMI (Z = 1.65, *p* = 0.10) (**Figure S1**), WHR (Z = 0.95, *p* = 0.34) (**Figure S2**), LDL-C (Z = 1.61, *p* = 0.11) (**Figure S3**), HDL-C, (Z = 1.60, *p* = 0.11) (**Figure S4**) or TG (Z = 1.06, *p* = 0.29) (**Figure S5**). Publication bias was observed in the association analyses between the rs1801282 polymorphism and WC (Z = 2.02, *p* = 0.04) (**Figure S6**) as well as TC (Z = 2.16, *p* = 0.03) (**Figure S7**). The trim-and-fill method was employed to adjust the publication bias, and the pooled results of both variables did not change after adjustment.

Publication bias was also evaluated for the association analyses between the rs3856806 polymorphism and obesity indexes as well as serum lipid variables, and no publication bias was detected for BMI (Z = 1.16, *p* = 0.24) (**Figure S8**), WC (Z = 0.23, *p* = 0.82) (**Figure S9**), WHR (Z = 1.31, *p* = 0.19) (**Figure S10**), TC (Z = 0.40, *p* = 0.69) (**Figure S11**), LDL-C (Z = 0.01, *p* = 0.99) (**Figure S12**), HDL-C (Z = 0.03, *p* = 0.98) (**Figure S13**) and TG (Z = 1.20, *p* = 0.23) (**Figure S14**).

## Discussion

PPARγ plays an essential role in the regulation of lipid metabolism. Being activated by endogenous and exogenous lipid ligands, PPARγ exerts its function as a transcription factor and mainly up-regulates the transcription of enzymes or transporters that play key roles in lipid metabolic pathways such as reverse cholesterol transport (143, 144), cholesterol transformation (143, 144), lipogenesis (145, 146), and fatty acid oxidation (147, 148). Therefore, variations in *PPARG* may lead to abnormal expression of this gene and/or dysfunction of PPARγ, resulting in aberrant expressions of PPARγ-targeted genes. The relationships between the rs1801282 and rs3856806 polymorphisms and CAD have been clarified by several previous meta-analyses (149–151). Wu et al. (149) performed a meta-analysis enrolled 22 studies and 23,375 subjects, and found that the GG genotype of the rs1801282 polymorphism conferred a higher risk of CAD than the CC genotype (OR = 1.30, 95% CI = 1.01 to 1.68, *p* = 0.04). Qian et al. (150) did a meta-analysis enrolled 9 studies and 3,878 subjects, and the results suggested that the T allele carriers of the rs3856806 polymorphism had a lower CAD risk than the CC homozygotes (OR = 0.69; 95% CI = 0.59 to 0.82, *p* < 0.001). Gonzlez-Castro et al. (151) expanded the sample size to 21 studies and 15,980 subjects, and confirmed Qian’s finding that the T allele of the rs3856806 polymorphism was a protective allele against CAD (OR = 0.33, 95% CI = 0.20 to 0.52, *p* < 0.001).

**Figure 3:**
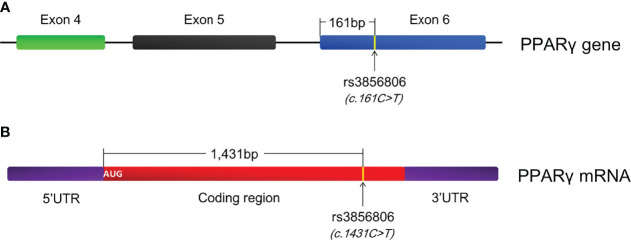
Annotation of the rs3856806 polymorphism in PPARγ gene. **(A)** c.161C>T is defined according to the position of this variant in exon 6 of PPARγ gene; **(B)** c.1431C>T is defined according to the position of this variant in PPARγ2 mRNA; PPARγ, peroxisome proliferator-activated receptor gamma.

The significant associations between the rs1801282 and rs3856806 polymorphisms and CAD prompted us to conduct the present meta-analysis to determine the relationships between these polymorphisms and obesity indexes as well as serum lipid levels since obesity and dyslipidemia are well-known risk factors for CAD (152–155). Indeed, this meta-analysis demonstrated that the G allele carriers of the rs1801282 polymorphism had significantly higher levels of BMI, WC and TC than the CC homozygotes; the T allele carriers of the rs3856806 polymorphism displayed lower levels of LDL-C, but higher levels of HDL-C than the CC homozygotes. These findings are in line with the previous meta-analyses which concluded that the G allele of the rs1801282 polymorphism was associated with an increased risk, while the T allele of the rs3856806 polymorphism was correlated with a reduced risk of CAD (149–151). To our knowledge, this is the first meta-analysis being done to date in the academic field to investigate the relationships of the rs1801282 and rs3856806 polymorphisms in PPARγ gene with obesity indexes, although there was a meta-analysis investigating the associations of the two polymorphisms with circulating lipid levels by Li and colleagues (156) in 2015. However, Li’s meta-analysis (156) mistakenly treated c.161C>T and c.1431C>T as two polymorphic loci. In fact, they are the same polymorphic locus with different names. c.161C>T was named according to the position of this variant in exon 6 of PPARγ gene since it is located at 161 bp downstream of the first nucleotide of exon 6 of *PPARG* (**Figure 3A**), and c.1431C>T was defined based on the position of this variant in PPARγ2 mRNA, as it is located at 1,431 bp downstream of the start genetic codon (**Figure 3B**). In addition, the present meta-analysis enrolled more studies (138 articles vs. 74 articles) and had larger sample size (78,652 vs.54,953), and thereby had a higher statistical power and more reliable results than Li’s meta-analysis (156).

In terms of the mechanisms underlying the associations between the rs1801282 and rs3856806 polymorphisms and obesity indexes as well as serum lipid levels, the first idea that comes to our mind is that the two polymorphisms lead to abnormal expression of *PPARG* and/or dysfunction of PPARγ, resulting in aberrant expressions of PPARγ-targeted genes. Indeed, Pihlajamäki et al. (157) examined the *PPARG* gene expression pattern of different genotypes of the rs1801282 polymorphism in human adipose tissues, and observed that the GG genotype was associated with a significantly higher mRNA expression level compared to the CC genotype. Other polymorphic loci in PPARγ gene have also been reported to modulate the gene expression of *PPARG*. The rs10865710 polymorphism (c.-681C>G) is located in the upstream promoter region of PPARγ3 gene and formed by a transversion from C to G. Lu et al. (158) observed that G allele of the rs10865710 polymorphism significantly reduced the DNA-binding activity of transcription factor CREB2 to PPARγ3 promoter. The rs948820149 polymorphism (c.-807A>C) is located in PPARγ2 promoter and C allele of this polymorphism was found to significantly down-regulate PPARγ2 expression by modulating the DNA-binding activity of transcription factor GRβ to PPARγ2 promoter (159). Another two promoter polymorphisms c.-1633C>T and c.-1572G>A in *PPARG* were also verified to regulate the expression efficiency of *PPARG* in Erhualian pigs (160). So far, there is no direct evidence that the *PPARG* polymorphisms affect the function of PPARγ.

Significant heterogeneity was detected in the association analyses between the rs1801282 polymorphism and obesity indexes as well as serum lipid levels. The outlier studies were identified by using Galbraith plots, and no significant changes in SMD values as well as their 95% CIs were found after excluding the outlier studies, which indicates that the associations between the rs1801282 polymorphism and the obesity indexes as well as serum lipid levels are robust. There are some limitations to the current study. First, this meta-analysis only enrolled the studies published in English and Chinese as it was difficult to get the full articles published in other languages. Second, the subgroup analyses were only conducted for ethnicities, health conditions, genders and ages of the subjects due to limitation on the amount of accessible data.

## Conclusions

The G allele carriers of the *PPARG* rs1801282 polymorphism had higher levels of BMI, WC and TC than the CC homozygotes; the T allele carriers of the *PPARG* rs3856806 polymorphism had lower levels of LDL-C and higher levels of HDL-C than the CC homozygotes; the effects of the *PPARG* rs1801282 and rs3856806 polymorphisms on obesity indexes and/or serum lipid levels are modulated by ethnicities, health conditions, genders and ages of the subjects.

## Data Availability Statement

The original contributions presented in the study are included in the article/**Supplementary Material**. Further inquiries can be directed to the corresponding author.

## Author Contributions

YS, SL, and CH conceived of the systematic review and meta-analysis, participated in the design, and drafted the manuscript. HN, QP, RW, and ZZ carried out the literature searches and collected the data. YS and SL performed the statistical analyses. All authors reviewed and approved the final manuscript.

## Funding

This project was supported by the Medical Science and Technology Project of Sichuan Provincial Health Commission [21PJ124], and the Scientific Research Project of Clinical Medical College and Affiliated Hospital of Chengdu University [Y2021010].

## Conflict of Interest

The authors declare that the research was conducted in the absence of any commercial or financial relationships that could be construed as a potential conflict of interest.

## Publisher’s Note

All claims expressed in this article are solely those of the authors and do not necessarily represent those of their affiliated organizations, or those of the publisher, the editors and the reviewers. Any product that may be evaluated in this article, or claim that may be made by its manufacturer, is not guaranteed or endorsed by the publisher.
