# Supplementary material for: G Allele of the rs1801282 Polymorphism in PPARγ Gene Confers an Increased Risk of Obesity and Hypercholesterolemia, While T Allele of the rs3856806 Polymorphism Displays a Protective Role Against Dyslipidemia: A Systematic Review and Meta-Analysis
Source: Front Endocrinol (Lausanne). 2022 Jun 29;13:919087. doi: 10.3389/fendo.2022.919087 (PMC9276935; doi:10.3389/fendo.2022.919087)
Supplement: Supplementary file 1 [file DataSheet_1.docx]

**Supplementary Figures S1-S14.**

**Figure S1.** Begg’s funnel plot of the association analysis between the rs1801282 polymorphism in *PPARG* and body mass index.

**Figure S2.** Begg’s funnel plot of the association analysis between the rs1801282 polymorphism in *PPARG* and waist-to-hip ratio.

**Figure S3.** Begg’s funnel plot of the association analysis between the rs1801282 polymorphism in *PPARG* and low-density lipoprotein cholesterol.

**Figure S4.** Begg’s funnel plot of the association analysis between the rs1801282 polymorphism in *PPARG* and high-density lipoprotein cholesterol.

**Figure S5.** Begg’s funnel plot of the association analysis between the rs1801282 polymorphism in *PPARG* and triglycerides.

**Figure S6.** Begg’s funnel plot of the association analysis between the rs1801282 polymorphism in *PPARG* and waist circumference.

**Figure S7.** Begg’s funnel plot of the association analysis between the rs1801282 polymorphism in *PPARG* and total cholesterol.

**Figure S8.** Begg’s funnel plot of the association analysis between the rs3856806 polymorphism in *PPARG* and body mass index.

**Figure S9.** Begg’s funnel plot of the association analysis between the rs3856806 polymorphism in *PPARG* and waist circumference.

**Figure S10.** Begg’s funnel plot of the association analysis between the rs3856806 polymorphism in *PPARG* and waist-to-hip ratio.

**Figure S11.** Begg’s funnel plot of the association analysis between the rs3856806 polymorphism in *PPARG* and total cholesterol.

**Figure S12.** Begg’s funnel plot of the association analysis between the rs3856806 polymorphism in *PPARG* and low-density lipoprotein cholesterol.

**Figure S13.** Begg’s funnel plot of the association analysis between the rs3856806 polymorphism in *PPARG* and high-density lipoprotein cholesterol.

**Figure S14.** Begg’s funnel plot of the association analysis between the rs3856806 polymorphism in *PPARG* and triglycerides.

**Figure S1.** Begg’s funnel plot of the association analysis between the rs1801282 polymorphism in *PPARG* and body mass index (Z = 1.65, *p* = 0.10).

**Figure S2.** Begg’s funnel plot of the association analysis between the rs1801282 polymorphism in *PPARG* and waist-to-hip ratio (Z = 0.95, *p* = 0.34).

**Figure S3.** Begg’s funnel plot of the association analysis between the rs1801282 polymorphism in *PPARG* and low-density lipoprotein cholesterol (Z = 1.61, *p* = 0.11).

**Figure S4.** Begg’s funnel plot of the association analysis between the rs1801282 polymorphism in *PPARG* and high-density lipoprotein cholesterol (Z = 1.60, *p* = 0.11).

**Figure S5.** Begg’s funnel plot of the association analysis between the rs1801282 polymorphism in *PPARG* and triglycerides (Z = 1.06, *p* = 0.29).

**Figure S6.** Begg’s funnel plot of the association analysis between the rs1801282 polymorphism in *PPARG* and waist circumference (Z = 2.02, *p* = 0.04).

**Figure S7.** Begg’s funnel plot of the association analysis between the rs1801282 polymorphism in *PPARG* and total cholesterol (Z = 2.16, *p* = 0.03).

**Figure S8.** Begg’s funnel plot of the association analysis between the rs3856806 polymorphism in *PPARG* and body mass index (Z = 1.16, *p* = 0.24).

**Figure S9.** Begg’s funnel plot of the association analysis between the rs3856806 polymorphism in *PPARG* and waist circumference (Z = 0.23, *p* = 0.82).

**Figure S10.** Begg’s funnel plot of the association analysis between the rs3856806 polymorphism in *PPARG* and waist-to-hip ratio (Z = 1.31, *p* = 0.19).

**Figure S11.** Begg’s funnel plot of the association analysis between the rs3856806 polymorphism in *PPARG* and total cholesterol (Z = 0.40, *p* = 0.69).

**Figure S12.** Begg’s funnel plot of the association analysis between the rs3856806 polymorphism in *PPARG* and low-density lipoprotein cholesterol (Z = 0.01, *p* = 0.99).

**Figure S13.** Begg’s funnel plot of the association analysis between the rs3856806 polymorphism in *PPARG* and high-density lipoprotein cholesterol (Z = 0.03, *p* = 0.98).

**Figure S14.** Begg’s funnel plot of the association analysis between the rs3856806 polymorphism in *PPARG* and triglycerides (Z = 1.20, *p* = 0.23).
