# Supplementary material for: G Allele of the rs1801282 Polymorphism in PPARγ Gene Confers an Increased Risk of Obesity and Hypercholesterolemia, While T Allele of the rs3856806 Polymorphism Displays a Protective Role Against Dyslipidemia: A Systematic Review and Meta-Analysis
Source: Front Endocrinol (Lausanne). 2022 Jun 29;13:919087. doi: 10.3389/fendo.2022.919087 (PMC9276935; doi:10.3389/fendo.2022.919087)
Supplement: Supplementary file 2 [file DataSheet_2.docx]

**Supplementary Tables S1-S8**

**Table S1.** Characteristics of the studies included in the meta-analysis for the rs1801282 polymorphism in *PPARG*.

**Table S2.** Characteristics of the studies included in the meta-analysis for the rs3856806 polymorphism in *PPARG*.

**Table S3.** Original data of the obesity indexes of the subjects with different genotypes of the rs1801282 polymorphism in *PPARG*.

**Table S4.** Original data of the serum lipid variables of the subjects with different genotypes of the rs1801282 polymorphism in *PPARG*.

**Table S5.** Original data of the obesity indexes of the subjects with different genotypes of the rs3856806 polymorphism in *PPARG*.

**Table S6.** Original data of the serum lipid variables of the subjects with different genotypes of the rs3856806 polymorphism in *PPARG*.

**Table S7.** The main contributors to the heterogeneity in the association analyses between the rs1801282 polymorphism in *PPARG* and obesity indexes as well as serum lipid variables.

**Table S8.** The main contributors to the heterogeneity in the association analyses between the rs3856806 polymorphism in *PPARG* and BMI as well as serum lipid variables.

**Table S1.** Characteristics of the studies included in the meta-analysis for the rs1801282 polymorphism in *PPARG*.

| **Authors, reference** | **Publication year** | **Study type** | **Ethnicity** | **Gender** | **Subjects** | **Age**  **(mean**±SD **or age range**) | **Outcomes** |
| --- | --- | --- | --- | --- | --- | --- | --- |
| Oh et al. ^[73]^ | 2000 | Cohort study | East Asian | M/F | Obesity patients/control subjects | 46.3±13.9 | WHR/BMI/TG/TC/LDL-C/HDL-C |
| Swarbrick et al. ^[38]^ | 2001 | Case-control study | Australian Caucasian | M/F | Obesity patients/control subjects | 52.98±12.88 | WHR/BMI/TG/TC/LDL-C/HDL-C |
| Mori et al. ^[74]^ | 2001 | Case-control study | East Asian | M/F | T2DM patients/control subjects | 64.59±11.65 | WHR/BMI/TG/TC |
| González et al. ^[42]^ | 2002 | Case-control study | European Caucasian | M/F | Obesity patients/control subjects | 48.69±8.68 | WHR/BMI/TG/TC/LDL-C/HDL-C |
| Schneider et al. ^[76]^ | 2002 | Cohort study | European Caucasian | M | T2DM patients/general population | 62.28±9.17 | BMI/TG/TC/LDL-C/HDL-C |
| Vaccaro et al. ^[77]^ | 2002 | Cross-sectional study | European Caucasian | M/F | Overweight patients/control subjects | 35-65 | WC/BMI/TG/HDL-C |
| Yamamoto et al. ^[78]^ | 2002 | Cohort study | East Asian | M/F | General population | 47.96±9.36 | BMI/TG/TC/LDL-C/HDL-C |
| Eriksson et al. ^[81]^ | 2003 | Cohort study | European Caucasian | M/F | General population | 69.60±2.83 | BMI/TG/TC/LDL-C/HDL-C |
| Iwata et al. ^[82]^ | 2003 | Cohort study | East Asian | M/F | T2DM patients | 61.01±8.60 | BMI/TG/TC/LDL-C/HDL-C |
| Niskanen et al. ^[83]^ | 2003 | Cohort study | European Caucasian | M/F | T2DM patients/control subjects | 54.89±5.53 | BMI |
| Robitaille et al. ^[84]^ | 2003 | Cohort study | American Caucasian | M/F | General population | 43.10±17.03 | WC/BMI |
| Baratta et al. ^[85]^ | 2003 | Cross-sectional study | European Caucasian | M/F | General population | 37.88±12.76 | BMI/TG/TC/HDL-C |
| Orio et al. ^[87]^ | 2004 | Case-control study | European Caucasian | F | PCOS/overweight/obesity patients/control subjects | 23.05±3.87 | WHR/BMI/TG/TC/LDL-C/HDL-C |
| Kim et al. ^[14]^ | 2004 | Cross-sectional study | East Asian | F | Overweight patients/general population | 27.06±6.48 | WHR/BMI/TG/TC/LDL-C/HDL-C |
| Tai et al. ^[6]^ | 2004 | Cross-sectional study | South Asian | M/F | T2DM/IGT patients/normal subjects | 48.00±12.59 | BMI/TG/TC/LDL-C/HDL-C |
| Pintérová et al. ^[88]^ | 2004 | Case-control study | European Caucasian | M/F | T2DM patients | 65.3±9.6 | BMI/TG/TC/LDL-C/HDL-C |
| Buzzetti et al. ^[89]^ | 2004 | Cross-sectional study | European Caucasian | M/F | General population | 42.65±13.54 | BMI/TG/TC/HDL-C |
| Andrulionytè et al. ^[43]^ | 2004 | Cohort study | Mix | M/F | T2DM patients | 54.7±7.9 | WHR/BMI/TG/TC/LDL-C/HDL-C |
| Pischon et al. ^[90]^ | 2005 | Case-control study | American Caucasian | M/F | Control subjects | 62.74±8.25 | BMI/TG/TC/LDL-C/HDL-C |
| Shen et al. ^[24]^ | 2005 | Case-control study | East Asian | M/F | CAD patients | 58.6±11.7 | BMI/TG/TC/LDL-C/HDL-C |
| Barbieri et al. ^[30]^ | 2005 | Cross-sectional study | European Caucasian | M/F | General population | 72±26 | BMI/TG/TC |
| Hahn et al. ^[91]^ | 2005 | Case-control study | European Caucasian | F | PCOS patients | 27±5.3 | BMI/TG/TC/LDL-C/HDL-C |
| Vänttinen et al. ^[92]^ | 2005 | Case-control study | European Caucasian | M/F | Obesity patients/control subjects | 32.03±10.19 | BMI/TG/TC/LDL-C/HDL-C |
| Mousavinasab et al. ^[93]^ | 2005 | Cohort study | European Caucasian | M | General population | 17-28 | WHR/BMI/TG/TC/LDL-C/HDL-C |
| Tavares et al. ^[44]^ | 2005 | Case-control study | South American | M/F | T2DM patients/control subjects | 53.80±12.03 | WHR/BMI/TG/TC/LDL-C/HDL-C |
| Zouari et al. ^[94]^ | 2005 | Cross-sectional study | African | M/F | T2DM patients | 51.7±11.7 | BMI/TG/TC |
| Danawati et al. ^[7]^ | 2005 | Case-control study | South Asian | M/F | T2DM patients/control subjects | 62.91±7.38 | BMI/TG/TC/HDL-C |
| Moon et al. ^[95]^ | 2005 | Case-control study | South Asian | M/F | Non-T2DM control subjects | 66±5 | WHR/BMI/TG/HDL-C |
| Buzzetti et al. ^[96]^ | 2005 | Cohort study | European Caucasian | M/F | Obesity patients | 10.25±2.17 | TG/TC/LDL-C/HDL-C |
| Li et al. ^[21]^ | 2006 | Case-control study | East Asian | M/F | MI patients/control subjects | 62.84±9.04 | WC/BMI/TG/TC |
| Stefański et al. ^[45]^ | 2006 | Cohort study | European Caucasian | M/F | Obesity/T2DM patients | 64.2±8.4 | WHR/BMI/TG/TC/LDL-C/HDL-C |
| Scaglioni et al. ^[97]^ | 2006 | Cohort study | European Caucasian | M/F | Obesity patients | 10.2±2.7 | TG/TC/LDL-C/HDL-C |
| Rhee et al. ^[98]^ | 2006 | Cross-sectional study | East Asian | F | General population | 51.46±6.74 | WC/BMI/TG/TC/LDL-C/HDL-C |
| Yilmaz et al. ^[99]^ | 2006 | Case-control study | West Asian | F | PCOS patients/control subjects | 23.87±6.78 | WC/WHR/BMI |
| Cardona et al. ^[100]^ | 2006 | Cross-sectional study | European Caucasian | M/F | MetS patients | 43.5±11.00 | WC/WHR/BMI/TG/TC/HDL-C |
| Rhee et al. ^[47]^ | 2007 | Cross-sectional study | East Asian | M/F | CAD patients/healthy subjects | 58.2±10.8 | BMI/TG/TC/LDL-C/HDL-C |
| Franks et al. ^[19]^ | 2007 | Randomized controlled trial | Mix | M/F | General population | 51.99±10.48 | WHR/BMI |
| Mattevi et al. ^[8]^ | 2007 | Cross-sectional study | South American | M | General population | 41.3±13.61 | WC/BMI/TG/TC/LDL-C/HDL-C |
| Dedoussis et al. ^[39]^ | 2007 | Cross-sectional study | European Caucasian | M/F | General population | 11-12 | WHR/BMI/TG/TC/HDL-C |
| Hamada et al. ^[31]^ | 2007 | Cohort study | East Asian | M/F | General population | 54±13 | BMI/TG/TC/LDL-C/HDL-C |
| Kim et al. ^[102]^ | 2007 | Human intervention study | East Asian | F | General population | 42.71±8.56 | WC/WHR/BMI |
| Helwig et al. ^[46]^ | 2007 | Cohort study | European Caucasian | M | General population | 58.98±5.49 | WC/WHR/BMI/TG/TC/LDL-C/HDL-C |
| Kotani et al. ^[103]^ | 2007 | Cohort study | East Asian | F | General population | 52.57±1.10 | BMI/TG/TC/HDL-C |
| Zafarmand et al. ^[104]^ | 2008 | Cohort study | European Caucasian | F | Healthy subjects | 57.18±6.08 | WHR/BMI/TC/LDL-C/HDL-C |
| Lu et al. ^[49]^ | 2008 | Case-control study | East Asian | M/F | General population | 94.6±4.0 | WHR/BMI/TG/TC/LDL-C/HDL-C |
| Montagnana et al. ^[51]^ | 2008 | Case-control study | European Caucasian | M/F | Hypertension/obesity patients | 57.65±5.91 | WC/TG/HDL-C |
| Mattevi et al. ^[8]^ | 2008 | Cross-sectional study | South American | F | General population | 38.7±16.19 | WC/BMI/TG/TC/LDL-C/HDL-C |
| Liu et al. ^[105]^ | 2008 | Cohort study | East Asian | M/F | General population | 49.96±13.95 | WC/WHR/BMI/TC |
| Badii et al. ^[106]^ | 2008 | Case-control study | West Asian | M/F | T2DM patients/control subjects | 50.14±11.48 | BMI |
| Li et al. ^[48]^ | 2008 | Cross-sectional study | East Asian | M/F | T2DM patients/control subjects | 51.72±15.34 | WC/WHR/BMI/TG/TC/LDL-C/HDL-C |
| Morini et al. ^[9]^ | 2008 | Cohort study | European Caucasian | M/F | General population | 36.59±11.85 | WC/BMI/TG/HDL-C |
| Yaffe et al. ^[10]^ | 2008 | Cohort study | African/American Caucasian | M/F | General population | 73.63±2.85 | WC/BMI/TG/HDL-C |
| Bendlová et al. ^[50]^ | 2008 | Case-control study | European Caucasian | M/F | General population | 32.15±11.42 | WC/WHR/BMI/TG/TC/LDL-C/HDL-C |
| Ramírez-Salazar et al. ^[15]^ | 2008 | Pharmacogenomics study | Mexican | F | Obesity patients/control subjects | 52±5 | WHR/BMI/TG/TC/LDL-C/HDL-C |
| Jorsal et al. ^[108]^ | 2008 | Case-control study | European Caucasian | M/F | Diabetic nephropathy patients | 42.2±10.4 | TC/HDL-C |
| Evangelisti et al. ^[109]^ | 2009 | Case-control study | European Caucasian | M/F | ACS patients/healthy subjects | 20-89 | BMI/TG/TC/LDL-C/HDL-C |
| Regieli et al. ^[110]^ | 2009 | Cohort study | European Caucasian | M | CAD patients | 55.7±7.97 | BMI/TG/TC/LDL-C/HDL-C |
| Ereqat et al. ^[22]^ | 2009 | Case-control study | West Asian | M/F | T2DM/obesity patients | 58.93±10.09 | BMI/TG/TC/LDL-C/HDL-C |
| Ben et al. ^[11]^ | 2009 | Case-control study | African | M/F | Obesity patients/control subjects | 45.77±11.45 | BMI/TG/TC/LDL-C/HDL-C |
| Milewicz et al. ^[112]^ | 2009 | Cohort study | European Caucasian | F | General population | 50-60 | WC/BMI/TG/TC/LDL-C/HDL-C |
| Mirzaei et al. ^[113]^ | 2009 | Cohort study | West Asian | M/F | Obesity/T2DM patients/control subjects | 36.49±18.43 | WC/BMI/TG/TC/HDL-C |
| Xita et al. ^[114]^ | 2009 | Case-control study | European Caucasian | F | PCOS patients | 26.6±6.9 | BMI/TG/TC/LDL-C/HDL-C |
| Haseeb et al. ^[71]^ | 2009 | Cross-sectional study | South Asian | M/F | General population | 61.88±10.79 | WC/WHR/BMI/TG/TC/HDL-C |
| Yue et al. ^[115]^ | 2009 | Case-control study | East Asian | M/F | Depression patients/control subjects | 93.50±3.35 | BMI/TG/TC/LDL-C/HDL-C |
| Koika et al. ^[116]^ | 2009 | Case-control study | European Caucasian | F | PCOS patients | 22.46±4.41 | BMI/TG/TC/LDL-C/HDL-C |
| Dedoussis et al. ^[117]^ | 2009 | Cohort study | European Caucasian | M/F | General population | 11.15±0.65 | WC/BMI/TG/TC/LDL-C/HDL-C |
| Fan et al. ^[16]^ | 2010 | Case-control study | East Asian | M/F | CAD patients/healthy subjects | 68.55±11.55 | WHR/BMI/TG/TC/LDL-C/HDL-C |
| Chistiakov et al. ^[53]^ | 2010 | Case-control study | European Caucasian | M/F | T2DM patients/control subjects | 60.95±9.00 | BMI/TG/TC/LDL-C/HDL-C |
| Bhagat et al. ^[17]^ | 2010 | Cross-sectional study | South Asian | M/F | Obesity patients | 44.53±15.64 | WC/WHR/BMI/TG/TC/HDL-C |
| Gao et al. ^[118]^ | 2010 | Case-control study | East Asian | M/F | Hypertension patients/control subjects | 53.22±15.98 | BMI/TG/TC/LDL-C/HDL-C |
| Chae et al. ^[52]^ | 2010 | Case-control study | East Asian | F | PCOS patients/control subjects | 27.31±4.88 | WHR/BMI/TG/TC/HDL-C |
| Zhang et al. ^[119]^ | 2010 | Human intervention study | East Asian | M/F | Dyslipidemia patients | 62.447±1.28 | WHR/BMI/TG/TC/HDL-C |
| de Kort et al. ^[120]^ | 2010 | Cohort study | American Caucasian | M/F | Short stature patients | 7.5±2.9 | TG/TC/LDL-C/HDL-C |
| Liu et al. ^[121]^ | 2010 | Case-control study | East Asian | M/F | T2DM patients | 64.37±11.7 | TC |
| Hsieh et al. ^[122]^ | 2010 | Case-control study | East Asian | M/F | T2DM patients | 57.86±11.46 | BMI/TC/LDL-C/HDL-C |
| Dongiovanni et al. ^[123]^ | 2010 | Case-control study | European Caucasian | M/F | NAFLD patients | 47.4±11 | HDL-C |
| Yilmaz-Aydogan et al. ^[40]^ | 2011 | Case-control study | West Asian | M/F | T2DM/CAD patients/healthy subjects | 57.78±11.88 | BMI/TG/TC/LDL-C/HDL-C |
| Bouchard-Mercier et al. ^[124]^ | 2011 | Cohort study | American Caucasian | M/F | General population | 37.89±11.35 | WC/BMI/TG/TC/LDL-C/HDL-C |
| Dedoussis et al. ^[125]^ | 2011 | Cohort study | European Caucasian | M/F | General population | 11.2±0.7 | WC/BMI |
| Jermendy et al. ^[54]^ | 2011 | Cohort study | European Caucasian | M/F | Obesity patients | 13.8±2.28 | WC/BMI/TG/TC/LDL-C/HDL-C |
| Garaulet et al. ^[55]^ | 2011 | Cohort study | European Caucasian | M/F | Overweight/obesity patients | 39.14±12.14 | WC/WHR/BMI/TG/TC/LDL-C/HDL-C |
| Ramakrishnan et al. ^[126]^ | 2011 | Cohort study | South Asian | M/F | General population | 23±5.5 | TG/TC/LDL-C/HDL-C |
| Passaro et al. ^[12]^ | 2011 | Cross-sectional study | European Caucasian | M/F | MetS patients | 55.51±13.21 | WC/BMI/TC/LDL-C/HDL-C |
| Chen et al. ^[127]^ | 2011 | Cross-sectional study | East Asian | M/F | Schizophrenia patients | 46.28±11.42 | WC/BMI/TG/HDL-C |
| Guan et al. ^[56]^ | 2011 | Case-control study | East Asian | M/F | OSAHS patients | 44.20±13.04 | WC/WHR/BMI/TG/TC/LDL-C/HDL-C |
| Estivalet et al. ^[128]^ | 2011 | Cohort study | South American | M/F | T2DM patients | 60.34±9.67 | WHR/BMI/TC/HDL-C |
| Bhatt et al. ^[129]^ | 2012 | Case-control study | South Asian | M/F | General population | 39.38±8.84 | WC/WHR/TG/TC/LDL-C/HDL-C |
| Hung et al. ^[28]^ | 2012 | Cross-sectional study | East Asian | M/F | HIV patients | 44.4±13.1 | TG/TC/LDL-C/HDL-C |
| Franck et al. ^[20]^ | 2012 | Cohort study | European Caucasian | M/F | T2DM patients | 60.73±2.97 | WC/BMI/TG/LDL-C/HDL-C |
| Fernández et al. ^[23]^ | 2012 | Cohort study | South American | M/F | MetS patients/normal controls | 38.96±10.04 | BMI/TG/TC/HDL-C |
| Youssef et al. ^[32]^ | 2013 | Case-control study | African | M/F | ACS patients/control subjects | 57.22±8.53 | TG |
| Curti et al. ^[130]^ | 2013 | Human intervention study | South American | M/F | General population | 56.6±11.63 | WC/TG/TC/LDL-C/HDL-C |
| Domenici et al. ^[57]^ | 2013 | Case-control study | South American | M/F | NAFLD patients | 44.5±2.5 | TG/TC/LDL-C/HDL-C |
| Yang et al. ^[131]^ | 2013 | Case-control study | East Asian | F | PCOS patients/control subjects | 27.86±6.17 | WC/WHR/BMI/TG/TC/LDL-C/HDL-C |
| Bhatt et al. ^[33]^ | 2013 | Case-control study | South Asian | M/F | Obesity/NAFLD patients | 37.63±6.96 | WC/BMI/TG/TC/LDL-C/HDL-C |
| Pei et al. ^[132]^ | 2013 | Case-control study | East Asian | M/F | T2DM patients | 25.15±2.77 | BMI/TG/TC/LDL-C/HDL-C |
| Arnaiz-Villena et al. ^[133]^ | 2013 | Cross-sectional study | Amerindian | M/F | General population | 38.15±9.28 | WC/TG/TC/HDL-C |
| Liu et al. ^[34]^ | 2014 | Case-control study | East Asian | M/F | Hemodialysis patients | 60.2±11.9 | BMI/TG/TC/LDL-C/HDL-C |
| Baldani et al. ^[58]^ | 2014 | Case-control study | European Caucasian | F | PCOS patients | 26.72±5.83 | WHR/BMI/TG/TC/LDL-C/HDL-C |
| Gu et al. ^[35]^ | 2014 | Cross-sectional study | East Asian | M/F | General population | 50.05±9.41 | TG/TC/LDL-C/HDL-C |
| Hsiao et al. ^[13]^ | 2015 | Case-control study | East Asian | M/F | General population | 43.13±12.13 | WC/BMI/TG/TC |
| Rocha et al. ^[135]^ | 2015 | Cohort study | South American | M/F | MetS patients | 57.9±6.83 | WC/BMI/TG/TC/LDL-C/HDL-C |
| Jiang et al. ^[26]^ | 2016 | Case-control study | East Asian | M/F | CAD patients/control subjects | 60.82±8.09 | TG/TC/LDL-C/HDL-C |
| Stryjecki et al. ^[59]^ | 2016 | Cohort study | Mexican | M/F | General population | 9.24±2.07 | WC/WHR/BMI/TG/TC/LDL-C/HDL-C |
| Rotter et al. ^[136]^ | 2016 | Cross-sectional study | European Caucasian | M | General population | 62±6.4 | WC/BMI/TG/TC/LDL-C/HDL-C |
| Li et al. ^[137]^ | 2016 | Case-control study | East Asian | M/F | Cerebral infarction | 66.0±14.0 | BMI/TG/TC/LDL-C/HDL-C |
| Grygiel-Gorniak et al. ^[69]^ | 2016 | Cross-sectional study | European Caucasian | F | General population | 59.31±5.46 | WC/WHR/BMI/TG/TC/LDL-C/HDL-C |
| Priya et al. ^[138]^ | 2016 | Pharmacogenomics study | South Asian | M/F | T2DM patients | 53.2±10.95 | WC/WHR/BMI |
| Aberle et al. ^[41]^ | 2016 | Cross-sectional study | European Caucasian | M/F | Obesity patients | 43.9±13.7 | BMI/TG/TC/LDL-C/HDL-C |
| Hasan et al. ^[27]^ | 2017 | Case-control study | African | M/F | CAD/T2DM patients | 48.77±9.73 | WC/BMI/TG/TC/LDL-C/HDL-C |
| Zaki et al. ^[18]^ | 2017 | Case-control study | African | F | PCOS patients/healthy controls | 23.45±2.46 | WC/WHR/BMI/TG/TC/LDL-C |
| Becer et al. ^[36]^ | 2017 | Case-control study | West Asian | M/F | Obesity patients/control subjects | 40.56±8.92 | WC/BMI/TG/TC/LDL-C/HDL-C |
| Zheng et al. ^[139]^ | 2018 | Randomized controlled trial | East Asian | M/F | T2DM patients | 59.92±9.91 | BMI/TG/TC/LDL-C/HDL-C |
| Rahimi et al. ^[37]^ | 2018 | Case-control study | West Asian | F | PCOS patients | 22.45±4.44 | TG/TC/LDL-C/HDL-C |
| Saeidi et al. ^[25]^ | 2018 | Case-control study | West Asian | M/F | Acne vulgaris patients/control subjects | 22.35±4.46 | TG/TC/LDL-C/HDL-C |
| Almeida et al. ^[60]^ | 2018 | Cross-sectional study | European Caucasian | M/F | Overweight/obesity patients/normal weight subjects | 9.79±0.60 | WC/WHR/BMI/TG/TC/LDL-C/HDL-C |
| Chmurzynska et al. ^[140]^ | 2019 | Human intervention study | European Caucasian | F | Central obesity patients | 60.43±4.47 | TG/TC/LDL-C/HDL-C |
| García-Ricobaraza et al. ^[141]^ | 2020 | Cohort study | European Caucasian | F | Pregnant women | 31.55±4.86 | TG/TC/LDL-C/HDL-C |
| Szkup et al. ^[61]^ | 2020 | Cross-sectional study | European Caucasian | F | General population | 54.3±4.2 | WHR/BMI/TG/TC/LDL-C/HDL-C |
| Carrillo-Venzor et al. ^[142]^ | 2020 | Cross-sectional study | Mexican | M/F | General population | 12-18 | TC/LDL-C |
| Vales-Villamarín et al. ^[29]^ | 2021 | Cross-sectional study | European Caucasian | M/F | General population | 7.2±0.6 | BMI/TG/TC/LDL-C/HDL-C |

*PPARG*, peroxisome proliferator-activated receptor gamma gene; SD, standard deviation; M, male; F, female; BMI, body mass index; WC, waist circumference; WHR, waist-to-hip ratio; TG: triglyceride; TC: total cholesterol; LDL-C: low-density lipoprotein cholesterol; HDL-C, high-density lipoprotein cholesterol; CAD, coronary artery disease; NAFLD, nonalcoholic fatty liver disease; ACS, acute coronary syndrome, T2DM, type 2 diabetes mellitus; IGT, impaired glucose tolerance; PCOS, polycystic ovarian syndrome; HIV, human immunodeficiency virus; MI, myocardial infarction; MetS, metabolic syndrome; OSAHS, Obstructive sleep apnea-hypopnea syndrome.

**Table S2.** Characteristics of the studies included in the meta-analysis for the rs3856806 polymorphism in *PPARG*.

| **Authors, reference** | **Publication year** | **Study type** | **Ethnicity** | **Gender** | **Subjects** | **Age**  **(mean**±SD **or age range**) | **Outcomes** |
| --- | --- | --- | --- | --- | --- | --- | --- |
| Meirhaeghe et al. ^[72]^ | 1998 | Cross-sectional study | European Caucasian | M/F | Obesity patients/control subjects | 35-64 | WHR/BMI/TG/TC |
| Wang et al. ^[67]^ | 1999 | Cohort study | Australian Caucasian | M/F | CAD patients/control subjects | 56.18±12.81 | WC/WHR/BMI/TG/TC/LDL-C/HDL-C |
| Peng et al. ^[75]^ | 2002 | Case-control study | East Asian | M/F | CAD patients/control subjects | 27-81 | BMI/TG/TC/LDL-C/HDL-C |
| Song et al. ^[79]^ | 2003 | Cross-sectional study | East Asian | M/F | Nephropathy patients | 36.9±13.1 | BMI |
| Arashiro et al. ^[80]^ | 2003 | Cross-sectional study | East Asian | M/F | Obesity patients | 9.5±0.1 | BMI/TG/TC/LDL-C/HDL-C |
| Chao et al. ^[86]^ | 2004 | Case-control study | East Asian | M | Heavy smokers | 31.59±5.43 | BMI/TG/TC/LDL-C/HDL-C |
| Maeda et al. ^[63]^ | 2004 | Case-control study | East Asian | M/F | T2DM patients | 64.0±12.7 | BMI/TG/TC/HDL-C |
| Tai et al. ^[6]^ | 2004 | Cross-sectional study | South Asian | M/F | T2DM/IGT/normal subjects | 48.00±12.59 | BMI/TG/TC/LDL-C/HDL-C |
| Tavares et al. ^[70]^ | 2005 | Case-control study | South American | M/F | T2DM patients/control subjects | 53.55±12.13 | WC/WHR/BMI/TG/TC/LDL-C/HDL-C |
| Moon et al. ^[95]^ | 2005 | Case-control study | South Asian | M/F | Non-T2DM control subjects | 66±5 | WHR/BMI/TG/HDL-C |
| Rhee et al. ^[98]^ | 2006 | Cross-sectional study | East Asian | F | General population | 51.46±6.74 | WC/BMI/TG/TC/LDL-C/HDL-C |
| Liu et al. ^[101]^ | 2007 | Case-control study | East Asian | M/F | CAD/ACS patients | 58.57±10.25 | TG/TC/LDL-C/HDL-C |
| Liu et al. ^[105]^ | 2008 | Cohort study | East Asian | M/F | General population | 49.96±13.95 | WC/WHR/BMI/TC |
| Morini et al. ^[9]^ | 2008 | Cohort study | European Caucasian | M/F | General population | 36.59±11.85 | WC/BMI/TG/HDL-C |
| Hui et al ^[107]^ | 2008 | Case-control study | East Asian | M/F | NAFLD patients/control subjects | 50.50±11.36 | WHR/BMI/TG/TC/LDL-C/HDL-C |
| Kotronen et al. ^[111]^ | 2009 | Cohort study | European Caucasian | M/F | General population | 20-75 | BMI/HDL-C |
| Evangelisti et al. ^[109]^ | 2009 | Case-control study | European Caucasian | M/F | ACS patients/healthy subjects | 20-89 | BMI/TG/TC/LDL-C/HDL-C |
| Haseeb et al. ^[136]^ | 2009 | Cross-sectional study | South Asian | M/F | General population | 61.88±10.79 | WC/WHR/BMI/TG/TC/HDL-C |
| Wan et al. ^[66]^ | 2010 | Cohort study | East Asian | M/F | CAD /T2DM patients | 62.04±11.81 | BMI/TG/TC/LDL-C/HDL-C |
| Yilmaz-Aydogan et al. ^[40]^ | 2011 | Case-control study | West Asian | M/F | T2DM/CAD patients/healthy subjects | 57.78±11.88 | BMI/TG/TC/LDL-C/HDL-C |
| Chen et al. ^[127]^ | 2011 | Cross-sectional study | East Asian | M/F | Schizophrenia patients | 46.28±11.42 | WC/BMI/TG/HDL-C |
| Hung et al. ^[28]^ | 2012 | Cross-sectional study | East Asian | M/F | HIV patients | 44.4±13.1 | TG/TC/LDL-C/HDL-C |
| Zhou et al. ^[62]^ | 2012 | Case-control study | East Asian | M/F | CAD patients/control subjects | 61.02±9.90 | BMI/TG/TC/LDL-C/HDL-C |
| Bhatt et al. ^[33]^ | 2013 | Case-control study | South Asian | M/F | Obesity/NAFLD patients/control subjects | 37.63±6.96 | WC/BMI/TG/TC/LDL-C/HDL-C |
| Liu et al. ^[34]^ | 2014 | Case-control study | East Asian | M/F | Hemodialysis patients | 60.2±11.9 | BMI/TG/TC/LDL-C/HDL-C |
| Gu et al. ^[35]^ | 2014 | Cross-sectional study | East Asian | M/F | General population | 50.05±9.41 | TG/TC/LDL-C/HDL-C |
| Chehaibi et al. ^[134]^ | 2014 | Case-control study | African | M/F | Stroke/T2DM patients | 63.52±11.45 | BMI/TG/TC/LDL-C/HDL-C |
| Chia et al. ^[68]^ | 2015 | Cohort study | Mix | M/F | General population | 53.3±14.15 | WC/WHR/BMI/TG/TC/LDL-C/HDL-C |
| Wei et al. ^[65]^ | 2016 | Case-control study | East Asian | M/F | Stroke patients/control subjects | 66.95±13.76 | TG/TC/LDL-C/HDL-C |
| Jiang et al. ^[26]^ | 2016 | Case-control study | East Asian | M/F | CAD patients/control subjects | 60.82±8.09 | TG/TC/LDL-C/HDL-C |
| Grygiel-Gorniak et al. ^[69]^ | 2016 | Cross-sectional study | European Caucasian | F | General population | 59.31±5.46 | WC/WHR/BMI/TG/TC/LDL-C/HDL-C |
| Rahimi et al. ^[37]^ | 2018 | Case-control study | West Asian | F | PCOS patients | 22.45±4.44 | TG/TC/LDL-C/HDL-C |
| Song et al. ^[64]^ | 2021 | Case-control study | East Asian | M/F | CAD patients/control subjects | 63.50±9.65 | BMI/TG/TC/LDL-C/HDL-C |

*PPARG*, peroxisome proliferator-activated receptor gamma gene; SD, standard deviation; M, male; F, female; BMI, body mass index; WC, waist circumference; WHR, waist-to-hip ratio; TG: triglycerides; TC: total cholesterol; LDL-C: low-density lipoprotein cholesterol; HDL-C, high-density lipoprotein cholesterol; CAD, coronary artery disease; NAFLD, nonalcoholic fatty liver disease; ACS, acute coronary syndrome, T2DM, type 2 diabetes mellitus; IGT, impaired glucose tolerance; PCOS, polycystic ovarian syndrome; HIV, human immunodeficiency virus.

**Table S3.** Original data of the obesity indexes of the subjects with different genotypes of the rs1801282 polymorphism in *PPARG*.

| **Authors, reference** | **Subjects** | **n** | | **BMI, kg/m^2^** | | **WC, cm** | | **WHR** | |
| --- | --- | --- | --- | --- | --- | --- | --- | --- | --- |
|  |  | CC | CG+GG | CC | CG+GG | CC | CG+GG | CC | CG+GG |
| Oh et al. ^[73]^ | Obesity/control subjects | 211 | 18 | 26.1±4.9 | 25.1±2.1 | - | - | 0.88±0.06 | 0.91±0.03 |
| Swarbrick et al. ^[38]^ | Obesity patients | 215 | 77 | 32.9±2.6 | 32.9±2.6 | - | - | 0.89±0.09 | 0.90±0.08 |
|  | Non-obesity control subjects | 277 | 94 | 22.0±1.8 | 22.1±2.0 | - | - | 0.81±0.08 | 0.81±0.08 |
| Mori et al. ^[74]^ | T2DM patients | 2041 | 103 | 23.2±3.6 | 23.3±4.1 | - | - | 0.89±0.08 | 0.93±0.09 |
|  | Normal control subjects | 1034 | 92 | 22.4±3.3 | 22.4±3.4 | - | - | - | - |
| González et al. ^[42]^ | Obesity males | 37 | 14 | 32.8±2.5 | 31.8±1.6 | - | - | 1.01±0.04 | 1.03±0.04 |
|  | Obesity females | 82 | 12 | 33.5±3.5 | 33.6±4.1 | - | - | 0.96±0.06 | 0.96±0.07 |
|  | All males | 174 | 36 | 27.37±3.83 | 28.07±3.49 | - | - | 0.99±0.07 | 1.00±0.04 |
|  | All females | 209 | 43 | 28.64±4.95 | 27.98±4.59 | - | - | 0.94±0.07 | 0.94±0.07 |
| Schneider et al. ^[76]^ | General population | 156 | 38 | 27.5±3.4 | 27.7±3.3 | - | - | - | - |
|  | T2DM patients | 87 | 13 | 28.7±4.1 | 27.6±3.4 | - | - | - | - |
| Vaccaro et al. ^[77]^ | Overweight patients | 137 | 33 | 29.9±4.3 | 30.5±4.4 | 100.0±12.4 | 101.6±8.9 | - | - |
|  | Non-overweight controls | 228 | 40 | 23.0±1.5 | 23.3±1.3 | 85.1±10.6 | 87.4±6.9 | - | - |
| Yamamoto et al. ^[78]^ | Males | 454 | 24 | 23.2±2.6 | 23.7±3.7 | - | - | - | - |
|  | Females | 109 | 8 | 20.8±2.9 | 19.8±2.6 | - | - | - | - |
| Eriksson et al. ^[81]^ | General population | 324 | 152 | 27.5±4.4 | 27.94±4.28 | - | - | - | - |
| Iwata et al. ^[82]^ | T2DM patients | 143 | 11 | 23.7±3.59 | 24.2±3.65 | - | - | - | - |
| Niskanen et al. ^[83]^ | T2DM patients | 56 | 14 | 30.4±5.1 | 30.7±5.9 | - | - | - | - |
|  | Non-T2DM control subjects | 93 | 26 | 27.1±4.6 | 27.1±2.9 | - | - | - | - |
| Robitaille et al. ^[84]^ | General population | 586 | 134 | 26.9±7.2 | 28.2±7.0 | 87.0±17.7 | 90.1±17.1 | - | - |
| Baratta et al. ^[85]^ | General population | 296 | 42 | 27±6 | 27±5 | - | - | - | - |
| Orio et al. ^[87]^ | PCOS patients | 113 | 7 | 30.3±5.5 | 30.2±5.4 | - | - | 0.88±0.4 | 0.88±0.5 |
|  | Non-PCOS control subjects | 115 | 5 | 30.0±5.4 | 30.1±5.3 | - | - | 0.86±0.3 | 0.86±0.4 |
| Kim et al. ^[14]^ | General population | 977 | 74 | 25.46±4.06 | 26.64±4.90 | - | - | 0.87±0.06 | 0.89±0.08 |
| Tai et al. ^[6]^ | T2DM patients | 374 | 46 | 26.99±7.74 | 27.48±5.70 | - | - | - | - |
|  | Impaired glucose tolerance | 499 | 39 | 25.66±7.60 | 25.78±4.81 | - | - | - | - |
|  | Normal control subjects | 2796 | 284 | 23.50±6.87 | 24.12±4.72 | - | - | - | - |
| Pintérová et al. ^[88]^ | T2DM patients | 99 | 34 | 31.2±4.9 | 31.5±5.8 | - | - | - | - |
| Buzzetti et al. ^[89]^ | General population | 1008 | 207 | 32.8±9 | 32.4±10 | - | - | - | - |
| Andrulionytè et al. ^[43]^ | T2DM patients | 592 | 178 | 30.65±4.00 | 31.11±4.45 | - | - | 0.92±0.08 | 0.93±0.08 |
| Pischon et al. ^[90]^ | Females | 386 | 99 | 25.5±3.93 | 25.3±3.98 | - | - | - | - |
|  | Males | 407 | 95 | 25.6±4.03 | 25.8±3.9 | - | - | - | - |
| Shen et al. ^[24]^ | CAD patients | 85 | 11 | 24.7±2.8 | 24.65±2.80 | - | - | - | - |
| Barbieri et al. ^[30]^ | General population | 362 | 67 | 25.0±2.9 | 23.5±2.6 | - | - | - | - |
| Hahn et al. ^[91]^ | PCOS patients | 79 | 22 | 30.0±9.4 | 30.0±7.6 | - | - | - | - |
| Vänttinen et al. ^[92]^ | Obesity patients | 38 | 14 | 32.4±4.2 | 32.1±2.8 | - | - | - | - |
|  | Non-obesity control subjects | 51 | 21 | 22.7±1.9 | 23.7±1.9 | - | - | - | - |
| Mousavinasab et al. ^[93]^ | General population | 173 | 79 | 26.3±3.8 | 26.49±5.00 | - | - | 0.91±0.07 | 0.91±0.08 |
| Tavares et al. ^[44]^ | T2DM patients | 171 | 36 | 30.6±7.3 | 30.5±5.7 | - | - | 0.93±0.11 | 0.96±0.14 |
|  | Healthy control subjects | 151 | 19 | 25.9±3.5 | 25.0±3.9 | - | - | 0.93±0.12 | 0.90±0.14 |
| Zouari et al. ^[94]^ | T2DM patients | 216 | 26 | 28.25±4.9 | 28.7±4.5 | - | - |  |  |
| Danawati et al. ^[7]^ | Non-T2DM control subjects | 196 | 7 | 22.6±3.6 | 26.7±4.6 | - | - | - | - |
|  | T2DM patients | 330 | 7 | 23.7±3.4 | 25.9±5.5 | - | - | - | - |
| Moon et al. ^[95]^ | Non-T2DM control subjects | 251 | 30 | 23.5±3.2 | 23.7±2.9 | - | - | 0.88±0.06 | 0.86±0.08 |
| Li et al. ^[21]^ | MI patients | 195 | 23 | 24.16±3.54 | 24.52±3.59 | 88.23±11.40 | 86.8±131.72 | - | - |
|  | Non-MI control subjects | 588 | 38 | 24.43±3.33 | 24.91±4.58 | 85.35±10.10 | 86.56±12.35 | - | - |
| Stefański et al. ^[45]^ | Obesity/T2DM patients | 154 | 60 | 34.0±3.7 | 34.6±3.8 | - | - | 0.97±0.07 | 0.97±0.07 |
| Rhee et al. ^[98]^ | General population | 226 | 27 | 23.99±2.93 | 24.98±2.86 | 78.47±8.34 | 79.52±8.10 | - | - |
| Yilmaz et al. ^[99]^ | PCOS patients | 85 | 15 | 24.70±6.78 | 21.78±6.65 | 83.50±16.63 | 76.38±15.72 | 0.84±0.07 | 0.80±0.07 |
|  | Non-PCOS control subjects | 78 | 22 | 25.98±6.46 | 22.07±4.17 | 82.37±12.56 | 75.67±10.69 | 0.81±0.08 | 0.78±0.07 |
| Cardona et al. ^[100]^ | MetS patients | 57 | 17 | 27.8±3.9 | 27.2±2.91 | 100.3±11.6 | 96.7±9.9 | 0.94±0.05 | 0.93±0.06 |
| Rhee et al. ^[47]^ | CAD/Healthy subjects | 243 | 24 | 25.6±2.7 | 24.4±3.6 | - | - | - | - |
| Franks et al. ^[19]^ | General population | 2787 | 569 | 34.0±6.8 | 34.2±6.4 | 105.0±14.7 | 107.0±14.3 | - | - |
| Mattevi et al. ^[8]^ | General population | 130 | 23 | 26.2±4.56 | 28.3±5.28 | 94.4±10.26 | 98.8±12.95 | - | - |
| Dedoussis et al. ^[39]^ | Males | 69 | 12 | 20.37±3.22 | 20.48±3.51 | - | - | 0.85±0.08 | 0.83±0.07 |
|  | Females | 79 | 13 | 20.76±3.86 | 19.23±2.96 | - | - | 0.80±0.06 | 0.78±0.10 |
| Hamada et al. ^[31]^ | General population | 353 | 26 | 24.1±4.6 | 24.4±5.5 | - | - | - | - |
| Kim et al. ^[102]^ | General population | 115 | 14 | 24.44±2.89 | 25.81±4.66 | 77.60±6.75 | 82.03±11.27 | 0.90±0.05 | 0.91±0.08 |
| Helwig et al. ^[46]^ | General population | 515 | 193 | 27.36±4.08 | 27.54±4.33 | 99.95±12.25 | 100.43±12.56 | 0.99±0.07 | 0.99±0.07 |
| Kotani K et al. ^[103]^ | General population | 310 | 25 | 22.7±3.52 | 23.9±4.00 | - | - | - | - |
| Zafarmand et al. ^[104]^ | Healthy subjects | 1143 | 376 | 25.8±3.9 | 25.85±4.06 | - | - | 0.78±0.05 | 0.78±0.48 |
| Lu et al. ^[49]^ | General population | 758 | 81 | 19±3.4 | 19±3.5 | - | - | 0.9±0.08 | 0.9±0.072 |
| Montagnana et al. ^[51]^ | Hypertension patients | 2847 | 929 | - | - | 86.2±13.2 | 86.1±13.6 | - | - |
|  | Obesity patients | 495 | 205 | - | - | 100.2±12.0 | 100.7±12.4 | - | - |
| Mattevi et al. ^[8]^ | General population | 153 | 29 | 25.7±4.95 | 25.5±4.31 | 86.6±12.37 | 85.0±10.77 | - | - |
| Liu et al. ^[105]^ | General population | 720 | 72 | 25.16±3.56 | 25.79±3.71 | 86.70±12.68 | 86.11±11.61 | 0.85±0.07 | 0.85±0.07 |
| Badii et al. ^[106]^ | T2DM patients | 361 | 39 | 30.6±5.0 | 31.15±5.88 | - | - | - | - |
|  | Non-T2DM control subjects | 401 | 49 | 27.4±4.3 | 28.52±5.50 | - | - | - | - |
| Li et al. ^[48]^ | T2DM patients | 202 | 39 | 25.27±4.46 | 27.47±4.76 | 89.99±18.08 | 99.49±13.71 | 0.91±0.08 | 0.97±0.11 |
|  | Non-T2DM control subjects | 236 | 56 | 25.68±4.42 | 26.65±5.11 | 88.63±11.61 | 90.12±12.38 | 0.88±0.06 | 0.89±0.37 |
| Morini et al. ^[9]^ | Males | 186 | 25 | 25.9±3.6 | 28.2±4.9 | 89.7±10.9 | 94.2±13.1 | - | - |
|  | Females | 315 | 40 | 24.9±4.8 | 24.7±5.0 | 78.9±11.3 | 77.2±11.0 | - | - |
| Yaffe et al. ^[10]^ | Africans | 1160 | 56 | 28.7±5.5 | 27.8±4.6 | 100.6±13.6 | 98.1±12.1 | - | - |
|  | American Caucasians | 1377 | 368 | 26.5±4.1 | 26.9±4.2 | 98.6±11.8 | 100.2±11.8 | - | - |
| Bendlová et al. ^[50]^ | Males | 72 | 26 | 24.90±3.53 | 24.31±3.72 | 85.61±9.61 | 85.70±10.81 | 0.85±0.07 | 0.86±0.06 |
|  | Females | 161 | 65 | 23.45±4.27 | 23.58±4.19 | 73.50±9.35 | 74.24±9.51 | 0.73±0.09 | 0.73±0.07 |
| Ramírez-Salazar et al. ^[15]^ | Obesity/Control subjects | 160 | 38 | 29±5 | 31±5.5 | - | - | 0.83±0.04 | 0.85±0.05 |
| Evangelisti et al. ^[109]^ | ACS/Healthy subjects | 427 | 71 | 26.7±3.9 | 25.83±4.05 | - | - | - | - |
| Regieli et al. ^[110]^ | CAD patients | 540 | 139 | 25.9±2.56 | 26.25±2.82 | - | - | - | - |
| Ereqat et al. ^[22]^ | T2DM patients | 179 | 23 | 31.5±6.2 | 31.5±6.7 | - | - | - | - |
|  | Obesity/T2DM patients | 106 | 15 | 35.4±4.9 | 35.1±4.9 | - | - | - | - |
| Ben et al. ^[11]^ | Obesity males | 151 | 21 | 34.55±4.45 | 37.67±5.86 | - | - | - | - |
|  | Obesity females | 197 | 18 | 43.24±5.68 | 43.14±7.40 | - | - | - | - |
| Milewicz et al. ^[112]^ | General population | 222 | 96 | 27.2±4.7 | 28.06±4.96 | 87±12 | 89.00±12.08 | - | - |
| Mirzaei et al. ^[113]^ | Obesity patients | 117 | 39 | 33.68±3.74 | 36.07±3.75 | 101.39±11.57 | 102.87±11.15 | - | - |
|  | Non-obesity control subjects | 134 | 22 | 25.27±2.96 | 27.21±1.95 | 86.84±10.47 | 92.78±11.42 | - | - |
|  | T2DM patients | 133 | 23 | 29.80±5.34 | 34.00±5.77 | 96.73±12.18 | 103.62±10.69 | - | - |
|  | Non-T2DM control subjects | 118 | 38 | 28.51±5.35 | 32.19±5.03 | 90.12±13.40 | 96.58±12.38 | - | - |
| Xita et al. ^[114]^ | PCOS patients | 150 | 30 | 28.2±7.6 | 26.6±6.9 | - | - | - | - |
| Haseeb et al. ^[71]^ | General population | 538 | 149 | 25.71±5.74 | 25.06±5.97 | 92.26±9.48 | 91.19±8.05 | 0.93±0.13 | 0.93±0.14 |
| Yue et al. ^[115]^ | Depression/Control subjects | 633 | 64 | 19.03±3.70 | 19.09±2.93 | - | - | - | - |
| Koika et al. ^[116]^ | PCOS patients | 136 | 20 | 25.76±6.69 | 24.40±3.49 | - | - | - | - |
| Dedoussis et al. ^[117]^ | Males | 313 | 61 | 20.4±3.4 | 19.7±3.4 | 70.7±9.6 | 68.7±9.5 | - | - |
|  | Females | 356 | 64 | 19.9±3.4 | 19.6±3.6 | 67.3±9.1 | 68.9±9.6 | - | - |
| Fan et al. ^[16]^ | CAD/Healthy subjects | 167 | 13 | 24.69±4.30 | 32.00±7.32 | - | - | 0.96±0.64 | 1.00±0.57 |
| Chistiakov et al. ^[53]^ | T2DM patients | 401 | 187 | 28.6±5.3 | 29.31±5.88 | - | - | - | - |
|  | Non-T2DM control subjects | 353 | 244 | 26.4±4.6 | 27.46±4.93 | - | - | - | - |
| Bhagat et al. ^[17]^ | Obesity patients | 326 | 18 | 27.1±7.7 | 38.0±10.5 | 86.0±19.5 | 108.1±20.6 | 0.88±0.14 | 1.01±0.25 |
| Gao et al. ^[118]^ | Hypertension patients | 337 | 8 | 25.05±3.09 | 25.31±2.85 | - | - | - | - |
|  | Non-hypertension controls | 131 | 6 | 23.61±2.86 | 22.69±3.89 | - | - | - | - |
| Chae et al. ^[52]^ | PCOS patients | 171 | 13 | 22.0±4.8 | 24.1±5.0 | - | - | 0.8±0.1 | 0.8±0.1 |
|  | Non-PCOS control subjects | 230 | 26 | 20.3±3.0 | 20.3±2.1 | - | - | 0.7±0.1 | 0.7±0.1 |
| Zhang et al. ^[119]^ | Dyslipidemia patients | 197 | 24 | 25.39±3.3 | 25.06±3.3 | - | - | 0.92±0.07 | 0.95±0.06 |
| Hsieh et al. ^[122]^ | T2DM patients | 197 | 53 | 26.6±4.2 | 26.6±3.6 | - | - | - | - |
| Yilmaz-Aydogan et al. ^[40]^ | CAD/T2DM patients | 89 | 13 | 26.35±4.06 | 26.18±3.99 | - | - | - | - |
|  | CAD patients | 87 | 13 | 26.65±3.15 | 25.98±3.23 | - | - | - | - |
|  | Healthy control subjects | 88 | 17 | 25.04±3.47 | 26.66±3.65 | - | - | - | - |
| Bouchard-Mercier et al. ^[124]^ | General population | 526 | 146 | 27.77±5.75 | 27.82±5.75 | 90.29±16.18 | 90.72±16.18 | - | - |
| Dedoussis et al. ^[125]^ | Males | 1155 | 203 | 17.41±2.88 | 17.32±2.73 | 56.48±10.66 | 56.46±10.03 | - | - |
|  | Females | 1148 | 184 | 17.35±2.88 | 17.48±3.09 | 56.26±9.78 | 57.94±10.48 | - | - |
| Jermendy et al. ^[54]^ | Obesity patients | 58 | 21 | 30.0±5.33 | 31.0±4.58 | 97.1±19.8 | 97.0±15.58 | - | - |
| Garaulet et al. ^[55]^ | Overweight/obesity patients | 1281 | 206 | 31.2±5.4 | 30.8±5.1 | 102.1±14.9 | 102.2±14.8 | 0.90±0.09 | 0.90±0.09 |
| Passaro et al. ^[12]^ | MetS males | 129 | 8 | 29.98±4.75 | 31.2±6.69 | 102.40±12.76 | 104.19±15.04 | - | - |
|  | MetS females | 205 | 22 | 30.98±6.19 | 34.02±6.16 | 95.92±14.05 | 98.61±13.38 | - | - |
| Chen et al. ^[127]^ | Schizophrenia males | 284 | 25 | 24.60±4.60 | 25.20±4.80 | 88.90±11.70 | 89.70±11.90 | - | - |
|  | Schizophrenia females | 266 | 25 | 25.60±4.90 | 26.50±4.50 | 86.40±13.00 | 90.40±12.10 | - | - |
| Guan et al. ^[56]^ | OSAHS patients | 315 | 42 | 27.27±3.51 | 26.91±3.10 | 97.48±9.05 | 96.37±6.68 | 0.95±0.05 | 0.95±0.05 |
| Estivalet et al. ^[128]^ | T2DM patients | 619 | 102 | 28.9±5.0 | 28.1±4.5 | - | - | 0.93±0.11 | 0.96±0.07 |
| Bhatt et al. ^[129]^ | General population | 409 | 86 | - | - | 86.3±12.0 | 86.85±11.78 | 0.90±0.09 | 0.89±0.08 |
| Franck et al. ^[20]^ | T2DM males | 243 | 66 | 29.4±3.9 | 30.1±5.1 | 104.2±11 | 107.4±14 | - | - |
|  | T2DM females | 134 | 39 | 30.7±5.3 | 32.2±6.3 | 102.2±14 | 102.8±14 | - | - |
| Fernández et al. ^[23]^ | MetS patients | 43 | 13 | 27.4±5.1 | 28.4±6.5 | 94.6±12.3 | 96.7±15.9 | - | - |
|  | Normal control subjects | 37 | 12 | 30.7±5.2 | 32.9±9.8 | 102.4±13.6 | 102.1±18.5 | - | - |
| Curti et al. ^[130]^ | General population | 111 | 23 | - | - | 99.7±12.59 | 99.3±11.26 | - | - |
| Yang et al. ^[131]^ | PCOS patients | 111 | 9 | 26.32±5.34 | 28.06±4.38 | 82.79±13.01 | 90.89±12.75 | 0.85±0.07 | 0.92±0.09 |
|  | Non-PCOS control subjects | 101 | 17 | 24.51±5.62 | 26.45±6.24 | 78.23±14.29 | 82.69±17.43 | 0.84±0.07 | 0.86±0.09 |
| Bhatt et al. ^[33]^ | Obesity/NAFLD patients | 124 | 38 | 28.0±2.8 | 28.9±4.87 | 93.9±8.8 | 96.28±11.4 | - | - |
|  | Obesity patients | 144 | 29 | 24.4±2.8 | 24.82±4.19 | 90.3±9.6 | 88.99±7.6 | - | - |
| Pei et al. ^[132]^ | T2DM patients | 60 | 7 | 25.29±2.79 | 23.91±2.39 | - | - | - | - |
| Arnaiz-Villena et al. ^[133]^ | General population | 257 | 65 | - | - | 88.49±11.30 | 89.30±12.16 | - | - |
| Liu et al. ^[34]^ | Hemodialysis patients | 89 | 10 | 21.5±3.5 | 22.3±3.7 | - | - | - | - |
| Baldani et al. ^[58]^ | PCOS patients | 106 | 45 | 23.7±4.2 | 22.6±4.1 | - | - | 0.79±0.08 | 0.78±0.07 |
| Hsiao et al. ^[13]^ | Females | 251 | 32 | 23.5±3.8 | 24.4±3.7 | 76.4±9.2 | 76.4±9.3 | - | - |
|  | Males | 355 | 25 | 25.0±3.5 | 25.4±4.3 | 86.2±8.6 | 86.9±11.1 | - | - |
| Rocha et al. ^[135]^ | MetS patients | 138 | 16 | 30.94±6.63 | 28.89±5.28 | 111.93±16.14 | 108.81±17.37 | - | - |
| Stryjecki et al. ^[59]^ | General population | 1067 | 377 | 19.67±4.17 | 19.67±4.30 | 66.57±11.71 | 66.42±11.97 | 0.85±0.06 | 0.85±0.06 |
| Rotter et al. ^[136]^ | General population | 186 | 86 | 28.25±4.16 | 27.67±4.09 | 101.64±11.95 | 100.5±11.11 | - | - |
| Li et al. ^[137]^ | Cerebral infarction | 274 | 28 | 23.58±2.94 | 23.44±3.35 | - | - | - | - |
| Grygiel-Gorniak et al. ^[69]^ | General population | 185 | 86 | 29.23±6.52 | 29.66±0.85 | 89.55±13.96 | 92.18±14.92 | 0.83±0.07 | 0.85±0.08 |
| Priya et al. ^[138]^ | T2DM patients | 24 | 6 | 26.70±3.79 | 24.46±3.02 | 91.33±8.75 | 87.17±11.74 | 0.90±0.07 | 0.90±0.06 |
| Aberle et al. ^[41]^ | Obesity patients | 361 | 101 | 29±7.9 | 28.7±7.2 | - | - | - | - |
| Hasan et al. ^[27]^ | CAD patients | 82 | 18 | 32.6±3.98 | 38±2.25 | 101±10.41 | 114.1±4.24 | - | - |
|  | T2DM patients | 92 | 13 | 30.8±4.41 | 32.2±1.62 | 93.8±18.22 | 100.2±5.80 | - | - |
|  | CAD/T2DM patients | 77 | 23 | 31.9±3.25 | 36.1±1.97 | 108.8±9.48 | 113.2±3.84 | - | - |
| Zaki et al. ^[18]^ | PCOS patients | 68 | 32 | 30.1±2.9 | 29.12±2.8 | 74.39 ±7.7 | 113.1±15.5 | 0.76±0.09 | 0.91±0.07 |
|  | Healthy control subjects | 96 | 24 | 24.2±8.9 | 23.12±7.8 | 85.39 ±7.7 | 90.1±9.5 | 0.86±0.09 | 0.81±0.07 |
| Becer et al. ^[36]^ | Obesity patients | 98 | 62 | 33.46±4.31 | 34.45±6.43 | 111.74 ±11.32 | 111.03±11.24 | - | - |
|  | Non-obesity control subjects | 81 | 59 | 20.64±3.67 | 21.57±2.58 | 83.75±8.59 | 85.37±8.34 | - | - |
| Zheng et al. ^[139]^ | T2DM patients | 133 | 17 | 25.17±3.83 | 24.68±3.11 | - | - | - | - |
| Almeida et al. ^[60]^ | Overweight/obesity patients | 64 | 12 | 22.04±2.16 | 21.42±1.18 | 72.70±7.68 | 72.54±4.87 | 0.92±0.06 | 0.91±0.04 |
|  | Normal weight subjects | 140 | 35 | 16.82±1.55 | 16.54±1.57 | 60.31±4.29 | 59.68±4.19 | 0.88±0.05 | 0.88±0.04 |
| Chmurzynska et al. ^[140]^ | Central obesity patients | 95 | 47 | - | - | 104.20±8.97 | 106.95±10.49 | - | - |
| Szkup et al. ^[61]^ | General population | 294 | 131 | 28.3±5.2 | 27.25±5.15 | 88.20±10.8 | 86.65±10.3 | 0.9±0.1 | 0.82±0.11 |
| Vales-Villamarín et al. ^[29]^ | Males | 535 | 98 | 16.9±2.4 | 17.08±2.46 | - | - | - | - |
|  | Females | 523 | 98 | 17.0±2.6 | 16.89±2.17 | - | - | - | - |

*PPARG*, peroxisome proliferator-activated receptor gamma gene; BMI, body mass index; WC, waist circumference; WHR, waist-to-hip ratio; T2DM, type 2 diabetes mellitus; PCOS, polycystic ovarian syndrome; CAD, coronary artery disease; MI, myocardial infarction; ACS, acute coronary syndrome; NAFLD, nonalcoholic fatty liver disease; MetS, metabolic syndrome; OSAHS, obstructive sleep apnea-hypopnea syndrome.

**Table S4.** Original data of the serum lipid variables of the subjects with different genotypes of the rs1801282 polymorphism in *PPARG*.

| **Authors, reference** | **Subjects** | **n** | | **TC, mmol/L** | | **LDL-C, mmol/L** | | **HDL-C, mmol/L** | | **TG, mmol/L** | |
| --- | --- | --- | --- | --- | --- | --- | --- | --- | --- | --- | --- |
|  |  | CC | CG+GG | CC | CG+GG | CC | CG+GG | CC | CG+GG | CC | CG+GG |
| Oh et al. ^[73]^ | Obesity/control subjects | 211 | 18 | 5.13±0.94 | 4.95±0.78 | 3.17±0.95 | 2.91±0.79 | 1.27±0.38 | 1.27±0.39 | 1.74±0.89 | 1.77±1.02 |
| Swarbrick et al. ^[38]^ | Obesity patients | 215 | 77 | 5.75±0.97 | 5.79±1.04 | 3.72±1.25 | 3.71±1.31 | 1.16±0.14 | 1.07±0.13 | 1.54±0.33 | 1.76±0.34 |
|  | Non-obesity control subjects | 277 | 94 | 5.38±1.21 | 5.24±1.19 | 3.50±0.91 | 3.37±0.81 | 1.50±0.38 | 1.50±0.44 | 1.02±0.57 | 0.98±0.46 |
| Mori et al. ^[74]^ | T2DM patients | 2041 | 103 | 5.25±1.01 | 5.66±1.19 | - | - | - | - | 1.60±1.52 | 1.50±0.94 |
| González et al. ^[42]^ | Obesity males | 37 | 14 | 5.85±0.99 | 6.16±0.99 | 3.89±0.75 | 4.18±0.88 | 1.11±0.27 | 1.23±0.23 | 2.0±1.65 | 1.64±0.69 |
|  | Obesity females | 82 | 12 | 5.90±1.14 | 5.77±1.35 | 3.95±1.04 | 3.87±1.27 | 1.36±0.29 | 1.40±0.34 | 1.31±0.58 | 1.08±0.35 |
|  | All males | 174 | 36 | 5.77±0.99 | 5.73±1.10 | 3.85±0.87 | 3.91±1.04 | 1.21±0.33 | 1.19±0.22 | 1.64±1.24 | 1.39±0.64 |
|  | All females | 209 | 43 | 5.75±1.05 | 5.56±1.01 | 3.76±0.98 | 3.62±0.88 | 1.47±0.33 | 1.53±0.36 | 1.17±0.57 | 0.91±0.36 |
| Schneider et al. ^[76]^ | General population | 156 | 38 | 5.4±1.3 | 5.2±1.2 | 3.8±1.1 | 3.6±1.0 | 1.0±0.3 | 1.0±0.3 | 1.8±1.5 | 1.6±0.9 |
|  | T2DM patients | 87 | 13 | 5.8±1.6 | 5.1±1.2 | 3.8±1.3 | 3.5±0.8 | 1.1±0.4 | 1.0±0.4 | 2.3±2.2 | 1.7±1.0 |
| Vaccaro et al. ^[77]^ | Overweight patients | 137 | 33 | - | - | - | - | 1.1±0.3 | 1.1±0.3 | 1.8±0.9 | 1.6±0.8 |
|  | Non-overweight controls | 228 | 40 | - | - | - | - | 1.3±0.4 | 1.3±0.4 | 1.3±0.6 | 1.5±1.0 |
| Yamamoto et al. ^[78]^ | Males | 454 | 24 | 5.17±0.70 | 5.33±0.64 | 3.23±0.69 | 3.44±0.61 | 1.41±0.34 | 1.32±0.24 | 1.36±0.77 | 1.39±0.76 |
|  | Females | 109 | 8 | 5.22±0.86 | 4.86±0.69 | 3.08±0.74 | 2.84±0.79 | 1.77±0.37 | 1.69±0.37 | 0.79±0.38 | 0.84±0.20 |
| Eriksson et al. ^[81]^ | General population | 324 | 152 | 5.9±1.1 | 6.12±1.19 | 3.8±0.9 | 3.92±1 | 1.44±1.35 | 1.45±1.36 | 1.29±1.52 | 1.33±1.58 |
| Iwata et al. ^[82]^ | T2DM patients | 143 | 11 | 5.26±0.96 | 4.97±0.80 | 3.12±0.96 | 2.90±0.86 | 1.41±0.36 | 1.44±0.46 | 1.63±1.08 | 1.33±0.80 |
| Baratta et al. ^[85]^ | General population | 296 | 42 | 5.2±1.1 | 5.2±1.0 | - | - | 1.2±0.4 | 1.2±0.3 | 1.2±0.7 | 1.1±0.6 |
| Orio et al. ^[87]^ | PCOS patients | 113 | 7 | 9.7±0.6 | 9.8±0.6 | 5.6±0.2 | 5.7±0.3 | 2.8±0.3 | 3.0±0.3 | 6.1±0.3 | 6.2±0.2 |
|  | Non-PCOS control subjects | 115 | 5 | 8.3±0.4 | 8.4±0.5 | 5.2±0.3 | 5.1±0.3 | 3.4±0.2 | 3.5±0.2 | 5.4±0.2 | 5.5±0.2 |
|  | Overweight patients | 423 | 33 | 4.71±0.79 | 4.72±0.97 | 2.92±0.73 | 3.02±0.71 | 1.23±0.31 | 1.24±0.29 | 1.24±0.49 | 1.23±0.56 |
| Tai et al. ^[6]^ | T2DM patients | 374 | 46 | 6.06±1.93 | 6.29±1.42 | 4.15±1.74 | 4.45±1.36 | 1.13±0.39 | 1.22±0.34 | 2.31±3.09 | 2.10±2.31 |
|  | Impaired glucose tolerance | 499 | 39 | 5.86±2.01 | 5.80±1.25 | 3.95±1.79 | 3.88±1.19 | 1.24±0.67 | 1.28±0.37 | 1.99±2.01 | 1.93±1.37 |
|  | Normal control subjects | 2796 | 284 | 5.43±1.59 | 5.45±1.18 | 3.49±1.59 | 3.51±1.01 | 1.31±0.53 | 1.35±0.34 | 1.43±1.59 | 1.36±1.01 |
| Pintérová et al. ^[88]^ | T2DM patients | 99 | 34 | 6.3±1.1 | 6.4±0.9 | 3.9±1.1 | 4.0±0.9 | 1.5±0.3 | 1.6±0.3 | 3.1±1.7 | 2.4±1.8 |
| Buzzetti et al. ^[89]^ | General population | 1008 | 207 | 5.31±1.11 | 5.29±1.13 | - | - | 1.12±0.37 | 1.14±0.39 | 1.3±0.82 | 1.25±0.84 |
| Andrulionytè et al. ^[43]^ | T2DM patients | 592 | 178 | 5.67±1.00 | 5.71±1.08 | 3.61±0.88 | 3.61±0.96 | 1.18±0.32 | 1.23±0.34 | 2.00±1.07 | 2.01±1.23 |
| Pischon et al. ^[90]^ | Females | 386 | 99 | 5.82±1.02 | 5.95±1.03 | 3.39±1.02 | 3.57±1.03 | 1.55±0.51 | 1.55±0.51 | 1.42±0.89 | 1.40±0.63 |
|  | Males | 407 | 95 | 5.28±1.04 | 5.4±0.76 | 3.26±1.04 | 3.39±0.76 | 1.19±0.52 | 1.19±0.25 | 1.74±1.37 | 1.71±1.21 |
| Shen et al. ^[24]^ | CAD patients | 85 | 11 | 4.23±1.01 | 5.91±1.71 | 2.87±0.63 | 3.9±0.97 | 1.34±0.31 | 0.91±0.24 | 1.01±0.38 | 1.36±0.77 |
| Barbieri et al. ^[30]^ | General population | 362 | 67 | 5.14±1.13 | 5.02±1.11 | - | - | - | - | 1.00±0.49 | 2.19±0.48 |
| Hahn et al. ^[91]^ | PCOS patients | 79 | 22 | 5.1±1.0 | 4.91±0.87 | 3.12±1.09 | 3.27±0.84 | 1.45±0.47 | 1.32±0.33 | 1.23±0.85 | 1.01±0.33 |
| Vänttinen et al. ^[92]^ | Obesity patients | 38 | 14 | 4.75±1.06 | 4.71±0.90 | 2.94±1.00 | 2.93±0.78 | 1.19±0.41 | 1.20±0.35 | 1.36±1.02 | 1.30±0.69 |
|  | Non-obesity control subjects | 51 | 21 | 4.45±0.80 | 4.25±0.88 | 2.72±0.73 | 2.49±0.86 | 1.27±0.26 | 1.34±0.39 | 1.04±0.52 | 1.00±0.60 |
| Mousavinasab et al. ^[93]^ | General population | 173 | 79 | 3.80±0.70 | 3.59±0.62 | 2.24±0.60 | 2.08±0.52 | 1.13±0.30 | 1.14±0.29 | 0.91±0.60 | 0.8±0.39 |
| Tavares et al. ^[44]^ | T2DM patients | 171 | 36 | 4.9±1.4 | 4.8±0.8 | 3.4±1.3 | 3.2±0.8 | 1.1±0.3 | 1.2±0.3 | 2.0±0.9 | 1.8±0.9 |
|  | Healthy control subjects | 151 | 19 | 4.7±0.7 | 4.4±0.6 | 3.3±0.8 | 2.8±0.6 | 1.2±0.3 | 1.3±0.3 | 1.5±0.7 | 1.4±1.0 |
| Zouari et al. ^[94]^ | T2DM patients | 216 | 26 | 4.99±0.78 | 5.09±0.93 | - | - | - | - | 1.30±0.79 | 1.24±0.45 |
|  | T2DM patients | 330 | 7 | 4.71±0.7 | 4.96±0.29 | - | - | 0.9±0.13 | 0.95±0.15 | 1.97±0.32 | 1.3±0.73 |
| Moon et al. ^[95]^ | Non-T2DM control subjects | 251 | 30 | - | - | - | - | 1.17±0.27 | 1.18±0.37 | 1.57±0.83 | 1.61±0.62 |
| Buzzetti et al. ^[96]^ | Obesity patients | 166 | 34 | 4.29±0.82 | 4.4±0.9 | 2.61±0.65 | 2.59±0.69 | 1.3±0.31 | 1.28±0.32 | 1.07±0.51 | 1.10±0.48 |
| Li et al. ^[21]^ | MI patients | 195 | 23 | 4.26±1.15 | 4.82±1.14 | - | - | - | - | 1.53±0.98 | 1.49±0.60 |
|  | Non-MI control subjects | 588 | 38 | 4.46±1.09 | 4.58±1.28 | - | - | - | - | 1.62±1.22 | 1.58±1.02 |
| Stefański et al. ^[45]^ | Obesity/T2DM patients | 154 | 60 | 5.55±1.23 | 5.52±1.31 | 3.36±1.03 | 3.35±1.05 | 1.35±0.33 | 1.28±0.32 | 1.73±0.95 | 1.77±0.83 |
| Scaglioni et al. ^[97]^ | Obesity patients | 114 | 26 | 4.19±0.67 | 4.35±0.65 | 2.35±0.63 | 2.46±0.55 | 1.29±0.27 | 1.29±0.28 | 1.14±0.64 | 1.29±0.83 |
| Rhee et al. ^[98]^ | General population | 226 | 27 | 5.19±0.93 | 5.30±1.08 | 3.03±0.85 | 3.14±1.08 | 1.53±0.36 | 1.53±0.39 | 1.37±0.77 | 1.37±0.78 |
| Cardona et al. ^[100]^ | MetS patients | 57 | 17 | 6.33±1.01 | 6.52±1.63 | - | - | 1.14±0.04 | 1.11±0.23 | 3.94±3.38 | 3.45±2.23 |
| Rhee et al. ^[47]^ | CAD/Healthy subjects | 243 | 24 | 5.0±1.1 | 4.8±1.0 | 2.9±0.8 | 2.7±0.9 | 1.3±0.3 | 1.3±0.3 | 1.8±1.1 | 2.0±1.4 |
| Mattevi et al. ^[8]^ | General population | 130 | 23 | 5.2±1.03 | 5.02±1.27 | 3.35±0.97 | 3.03±0.83 | 1.05±0.27 | 1.05±0.22 | 1.81±1.79 | 2.12±0.39 |
| Dedoussis et al. ^[39]^ | Males | 69 | 12 | 4.86±0.73 | 4.86±0.60 | 3.21±0.61 | 3.35±0.42 | 1.22±0.20 | 1.11±0.22 | 0.87±0.27 | 0.86±0.19 |
|  | Females | 79 | 13 | 5.10±0.81 | 4.66±0.71 | 3.33±0.70 | 3.13±0.67 | 1.20±0.26 | 1.16±0.11 | 1.06±0.35 | 0.86±0.13 |
| Hamada et al. ^[31]^ | General population | 353 | 26 | 5.66±0.97 | 5.61±0.96 | 2.86±0.75 | 2.77±0.70 | 1.60±0.37 | 1.64±0.46 | 3.20±2.27 | 3.78±3.55 |
| Helwig et al. ^[46]^ | General population | 515 | 193 | 5.84±1.04 | 5.94±1.14 | 3.73±0.91 | 3.74±0.92 | 1.38±0.39 | 1.4±0.4 | 1.58±0.91 | 1.61±1.41 |
| Kotani K et al. ^[103]^ | General population | 310 | 25 | 5.72±0.88 | 5.72±0.95 | - | - | 1.67±0.35 | 1.64±0.45 | 1.25±0.88 | 1.74±1.55 |
| Zafarmand et al. ^[104]^ | Healthy subjects | 1143 | 376 | 5.9±1 | 5.81±1.01 | 3.9±0.9 | 3.91±0.92 | 1.6±0.4 | 1.59±0.4 | - | - |
| Lu et al. ^[49]^ | General population | 758 | 81 | 4.15±0.817 | 4.12±0.8 | 2.25±0.59 | 2.24±0.63 | 1.53±0.35 | 1.58±0.33 | 1.25±0.61 | 1.03±0.5 |
| Montagnana et al. ^[51]^ | Hypertension patients | 2847 | 929 | - | - | - | - | 1.3±0.4 | 1.4±0.4 | 1.5±0.8 | 1.4±0.7 |
|  | Obesity patients | 495 | 205 | - | - | - | - | 1.2±0.3 | 1.2±0.3 | 1.8±0.9 | 1.7±0.9 |
| Mattevi et al. ^[8]^ | General population | 153 | 29 | 4.93±1.06 | 4.86±0.96 | 3.05±0.9 | 3.11±0.91 | 1.23±0.32 | 1.19±0.28 | 1.4±1.06 | 1.24±0.77 |
| Liu et al. ^[105]^ | General population | 720 | 72 | 5.24±1.09 | 5.07±1.18 | - | - | - | - | - | - |
| Li et al. ^[48]^ | T2DM patients | 202 | 39 | 4.44±1.78 | 4.46±1.35 | 2.68±1.23 | 2.68±1.47 | 0.98±0.68 | 1.29±1.56 | 2.14±1.79 | 2.78±2.73 |
|  | Non-T2DM control subjects | 236 | 56 | 4.55±1.16 | 4.57±1.01 | 2.93±1.19 | 2.95±1.24 | 1.11±0.48 | 1.09±1.43 | 1.86±5.15 | 1.63±1.31 |
| Morini et al. ^[9]^ | Males | 186 | 25 | - | - | - | - | 1.21±0.32 | 1.11±0.29 | 1.12±0.76 | 1.47±0.82 |
|  | Females | 315 | 40 | - | - | - | - | 1.48±0.32 | 1.55±0.34 | 0.85±0.44 | 0.86±0.50 |
| Yaffe et al. ^[10]^ | Africans | 1160 | 56 | - | - | - | - | 1.41±0.41 | 1.47±0.44 | 1.21±0.51 | 1.15±0.38 |
|  | American Caucasians | 1377 | 368 | - | - | - | - | 1.28±0.39 | 1.28±0.39 | 1.52±0.73 | 1.53±0.75 |
| Bendlová et al. ^[50]^ | Males | 72 | 26 | 4.60±1.16 | 4.64±0.95 | 2.70±1.16 | 2.70±0.94 | 1.24±0.36 | 1.30±0.08 | 1.06±0.65 | 1.10±0.72 |
|  | Females | 161 | 65 | - | - | - | - | 1.57±0.54 | 1.53±0.55 | - | - |
| Ramírez-Salazar et al. ^[15]^ | Obesity/Control subjects | 160 | 38 | 4.81±0.98 | 4.60±0.72 | 3.08±0.91 | 2.95±0.75 | 0.96±0.18 | 0.98±0.26 | 1.68±0.88 | 1.52±0.70 |
| Jorsal et al. ^[108]^ | Diabetic nephropathy patients | 290 | 125 | 5.6±1.2 | 5.54±1.21 | - | - | 1.52±0.70 | 1.43±0.49 | - | - |
| Evangelisti et al. ^[109]^ | ACS/Healthy subjects | 427 | 71 | 5.1±1.13 | 4.99±1.05 | 2.9±0.82 | 2.85±0.82 | 1.43±0.35 | 1.41±0.33 | 1.32±0.89 | 1.36±0.80 |
| Regieli et al. ^[110]^ | CAD patients | 540 | 139 | 6.05±0.93 | 6.02±0.9 | 4.31±0.7 | 4.03±0.78 | 0.93±0.23 | 0.91±0.22 | 1.78±0.7 | 1.85±0.79 |
| Ereqat et al. ^[22]^ | T2DM patients | 179 | 23 | 4.98±1.32 | 5.46±1.3 | 2.97±1.23 | 3.21±1.13 | 1.10±0.4 | 1.13±0.35 | 1.99±0.93 | 1.96±0.81 |
|  | Obesity/T2DM patients | 106 | 15 | 5.10±1.36 | 6.00±1.08 | 3.03±1.27 | 3.67±0.97 | 1.14±0.42 | 1.08±0.39 | 2.05±0.89 | 2.00±0.91 |
| Ben et al. ^[11]^ | Obesity males | 151 | 21 | 5.05±1.06 | 4.84±0.71 | 3.13±0.94 | 3.03±0.60 | 1.06±0.23 | 1.12±0.25 | 1.85±0.97 | 1.48±0.58 |
|  | Obesity females | 197 | 18 | 4.70±0.91 | 5.09±0.88 | 2.79±0.79 | 3.10±0.78 | 1.26±0.31 | 1.35±0.32 | 1.33±0.67 | 1.58±0.86 |
|  | Non-obesity control subjects | 271 | 17 | 4.64±0.97 | 4.52±0.81 | 2.74±0.87 | 2.73±0.78 | 1.37±0.37 | 1.29±0.40 | 1.13±0.61 | 1.07±0.44 |
| Milewicz et al. ^[112]^ | General population | 222 | 96 | 6.29±1.05 | 6.47±1.17 | 3.93±1.00 | 4.07±1.06 | 1.83±0.44 | 1.82±0.47 | 1.21±0.54 | 1.27±0.59 |
| Mirzaei et al. ^[113^ | Obesity patients | 117 | 39 | 5.51±1.07 | 5.50±0.82 | - | - | 1.66±0.29 | 1.66±0.36 | 2.54±2.06 | 2.42±1.62 |
|  | Non-obesity control subjects | 134 | 22 | 5.10±1.35 | 5.03±1.43 | - | - | 1.57±0.49 | 1.55±0.47 | 2.13±1.74 | 3.19±1.54 |
|  | T2DM patients | 133 | 23 | 5.40±1.25 | 5.49±0.91 | - | - | 1.65±0.39 | 1.62±0.31 | 2.79±2.25 | 3.77±2.43 |
|  | Non-T2DM control subjects | 118 | 38 | 5.17±1.23 | 5.22±1.19 | - | - | 1.57±0.43 | 1.62±0.46 | 1.79±1.23 | 2.05±1.38 |
| Xita et al. ^[114]^ | PCOS patients | 150 | 30 | 4.63±0.85 | 4.94±0.89 | 4.19±0.77 | 4.44±0.78 | 1.21±0.36 | 1.32±0.35 | 0.95±0.58 | 1.07±0.73 |
| Haseeb et al. ^[71]^ | General population | 538 | 149 | 6.27±5.21 | 7.33±7.10 | - | - | 1.00±0.48 | 1.02±0.50 | 2.11±1.10 | 2.20±1.07 |
| Yue et al. ^[115]^ | Depression/Control subjects | 633 | 64 | 4.15±0.85 | 4.10±0.84 | 2.29±0.99 | 2.20±0.59 | 1.58±0.59 | 1.56±0.58 | 1.21±0.63 | 1.36±0.89 |
| Koika et al. ^[116]^ | PCOS patients | 136 | 20 | 5.00±1.22 | 4.87±1.15 | 3.05±1.07 | 2.77±0.99 | 1.44±0.32 | 1.72±0.55 | 1.07±0.56 | 0.83±0.42 |
| Dedoussis et al. ^[117]^ | Males | 313 | 61 | 4.91±0.70 | 4.83±0.70 | 3.21±0.57 | 3.11±0.60 | 1.38±0.26 | 1.38±0.26 | 0.68±0.24 | 0.75±0.30 |
|  | Females | 356 | 64 | 4.77±0.73 | 4.69±0.73 | 3.08±0.59 | 3.06±0.57 | 1.33±0.25 | 1.32±0.28 | 0.75±0.25 | 0.75±0.30 |
| Fan et al. ^[16]^ | CAD/Healthy subjects | 167 | 13 | 4.87±1.29 | 5.93±2.57 | 2.87±0.98 | 3.29±1.10 | 1.13±0.46 | 1.06±0.32 | 1.88±1.19 | 2.01±0.61 |
| Chistiakov et al. ^[53]^ | T2DM patients | 401 | 187 | 4.9±1.2 | 5.18±1.4 | 3.4±1.2 | 2.99±1.01 | 1.3±0.3 | 1.28±0.39 | 2.5±0.6 | 1.66±0.54 |
|  | Non-T2DM control subjects | 353 | 244 | 4.9±1.1 | 5.27±1.20 | 3.2±1.0 | 3.33±1.16 | 1.2±0.3 | 1.29±0.3 | 1.8±0.5 | 1.39±0.4 |
| Bhagat et al. ^[17]^ | Obesity patients | 326 | 18 | 5.16±1.24 | 5.7±1.06 | - | - | 1.11±0.16 | 1.06±0.15 | 1.85±0.78 | 2.52±0.78 |
| Gao et al. ^[118]^ | Hypertension patients | 337 | 8 | 4.93±0.89 | 5.18±1.24 | 1.26±0.37 | 1.22±0.47 | 3.07±0.80 | 2.80±0.84 | 1.90±1.44 | 2.44±2.04 |
|  | Non-hypertension controls | 131 | 6 | 4.73±0.84 | 5.13±1.66 | 1.39±0.45 | 1.52±0.35 | 2.79±0.63 | 2.93±0.81 | 1.63±1.18 | 1.47±1.17 |
| Chae et al. ^[52]^ | PCOS patients | 171 | 13 | 4.53±0.93 | 4.49±0.72 | - | - | 1.49±0.39 | 2.13±0.33 | - | - |
|  | Non-PCOS control subjects | 230 | 26 | 4.36±0.69 | 4.61±0.60 | - | - | 1.65±0.33 | 1.65±0.41 | - | - |
| Zhang et al. ^[119]^ | Dyslipidemia patients | 197 | 24 | 5.28±0.94 | 5.44±1.37 | - | - | 1.13±0.42 | 1.07±0.43 | 2.70±1.86 | 2.55±1.11 |
| de Kort et al. ^[120]^ | Short stature patients | 168 | 70 | 4.3±0.8 | 4.2±0.7 | 2.4±0.7 | 2.4±0.6 | 1.4±0.4 | 1.5±0.4 | 0.8±0.3 | 0.8±0.5 |
| Liu L et al. ^[121^ | T2DM patients | 698 | 62 | 4.9±1.3 | 5.1±1.4 | - | - | - | - | - | - |
| Hsieh et al. ^[122]^ | T2DM patients | 197 | 53 | 4.98±1.22 | 4.69±1.27 | 3.05±1.37 | 2.86±1.1 | 1.27±0.41 | 1.15±0.28 | - | - |
| Dongiovanni et al. ^[123]^ | NAFLD patients | 166 | 36 | - | - | - | - | 1.28±0.36 | 1.31±0.36 | - | - |
| Yilmaz-Aydogan et al. ^[40]^ | CAD/T2DM patients | 89 | 13 | 5.04±1.43 | 4.99±1.12 | 3.08±1.09 | 3.06±1.01 | 0.96±0.24 | 1.03±0.22 | 1.79±1.17 | 1.85±0.67 |
|  | CAD patients | 87 | 13 | 5.55±1.52 | 5.85±1.17 | 3.58±1.20 | 3.74±1.06 | 1.03±0.19 | 1.14±0.13 | 1.53±0.74 | 1.51±0.51 |
|  | Healthy control subjects | 88 | 17 | 4.96±1.39 | 4.84±1.66 | 3.26±1.28 | 3.23±1.38 | 1.17±0.21 | 0.93±0.27 | 1.62±0.96 | 2.08±0.95 |
| Bouchard-Mercier et al. ^[124]^ | General population | 526 | 146 | 4.58±1.00 | 4.61±1.01 | 2.89±0.95 | 2.89±0.95 | 1.36±0.36 | 1.39±0.36 | 1.15±0.36 | 1.12±0.36 |
| Jermendy et al. ^[54]^ | Obesity patients | 58 | 21 | 4.09±0.69 | 4.37±0.78 | 2.70±0.76 | 2.86±0.73 | 1.43±0.38 | 1.49±0.37 | 1.35±0.69 | 1.37±0.87 |
| Garaulet et al. ^[55]^ | Overweight/obesity patients | 1281 | 206 | 5.0±0.9 | 4.9±0.9 | 3.0±0.8 | 3.0±0.8 | 1.4±0.4 | 1.4±0.4 | 1.2±0.6 | 1.1±0.5 |
| Ramakrishnan et al. ^[126]^ | General population | 916 | 245 | 4.99±1.08 | 5.05±1.15 | 3.05±0.88 | 3.10±1.00 | 1.22±0.31 | 1.23±0.29 | 1.54±0.93 | 1.57±0.91 |
| Passaro et al. ^[12]^ | MetS males | 129 | 8 | 5.5±15 | 5.5±1.0 | 3.4±1.4 | 3.4±0.9 | 1.2±0.3 | 1.4±0.3 | - | - |
|  | MetS females | 205 | 22 | 5.7±1.3 | 6.1±1.1 | 3.6±1.2 | 4.0±1.1 | 1.4±0.3 | 1.4±0.4 | - | - |
| Chen et al. ^[127]^ | Schizophrenia males | 284 | 25 | - | - | - | - | 1.18±0.34 | 1.11±0.30 | 1.78±1.47 | 1.80±1.32 |
|  | Schizophrenia females | 266 | 25 | - | - | - | - | 1.37±0.42 | 1.37±0.36 | 1.52±0.96 | 1.64±0.92 |
| Guan et al. ^[56]^ | OSAHS patients | 315 | 42 | 4.85±0.94 | 5.07±1.05 | 3.26±0.83 | 3.11±0.77 | 1.06±0.26 | 1.13±0.25 | 2.15±1.14 | 2.36±2.33 |
| Estivalet et al. ^[128]^ | T2DM patients | 619 | 102 | 5.4±1.2 | 5.4±1.4 | - | - | 1.2±0.3 | 1.2±0.3 | - | - |
| Bhatt et al. ^[129]^ | General population | 409 | 86 | 4.78±1.04 | 4.66±1.03 | 2.96±0.93 | 2.86±0.89 | 1.09±0.28 | 1.09±0.31 | 1.63±0.92 | 1.58±0.79 |
| Hung et al. ^[28]^ | HIV patients | 83 | 8 | 5.28±1.22 | 5.77±0.78 | 2.88±0.89 | 3.57±0.48 | 1.17±0.30 | 1.20±0.31 | 3.79±4.22 | 2.75±1.28 |
| Franck et al. ^[20]^ | T2DM males | 243 | 66 | - | - | 2.59±0.71 | 2.58±0.73 | 1.27±0.34 | 1.23±0.28 | 1.76±1.2 | 1.90±1.0 |
|  | T2DM females | 134 | 39 | - | - | 2.76±0.90 | 2.78±0.65 | 1.39±0.30 | 1.52±0.50 | 1.74±0.92 | 1.77±0.86 |
| Fernández et al. ^[23]^ | MetS patients | 43 | 13 | 4.34±1.02 | 5.09±1.05 | - | - | 0.88±0.25 | 0.92±0.23 | 1.48±0.84 | 2.27±1.81 |
|  | Normal control subjects | 37 | 12 | 4.16±0.96 | 3.47±0.53 | - | - | 0.99±0.29 | 1.05±0.22 | 1.44±0.78 | 0.88±0.42 |
| Youssef et al. ^[32]^ | ACS patients | 181 | 58 | - | - | - | - | - | - | 1.79±0.72 | 1.61±0.83 |
|  | Non-ACS control subjects | 158 | 86 | - | - | - | - | - | - | 1.06±0.46 | 1.21±0.48 |
| Curti et al. ^[130]^ | General population | 111 | 23 | 5.22±1.58 | 4.84±1.14 | 3.32±1.53 | 2.98±1.03 | 1.06±0.38 | 1.02±0.25 | 1.82±1.12 | 1.79±0.84 |
| Domenici et al. ^[57]^ | NAFLD patients | 87 | 16 | 5.09±1.06 | 5.47±1.17 | 3.79±1.78 | 3.09±0.86 | 1.11±0.26 | 1.16±0.24 | 2.05±1.21 | 1.99±0.88 |
| Yang et al. ^[131]^ | PCOS patients | 111 | 9 | 4.47±1.00 | 4.92±0.97 | 2.64±0.83 | 2.77±0.71 | 1.45±0.39 | 1.31±0.50 | 1.27±0.74 | 2.00±0.97 |
|  | Non-PCOS control subjects | 101 | 17 | 4.44±1.49 | 3.59±1.60 | 2.51±0.71 | 2.57±0.51 | 1.57±0.60 | 1.50±0.33 | 1.01±0.60 | 1.28±1.16 |
| Bhatt et al. ^[33]^ | Obesity/NAFLD patients | 124 | 38 | 4.84±0.76 | 5.05±0.91 | 2.80±0.56 | 2.83±0.58 | 1.00±0.004 | 0.99±0.04 | 1.92±0.86 | 2.04±0.79 |
|  | Obesity patients | 144 | 29 | 4.64±0.70 | 4.71±0.64 | 2.71±0.58 | 2.66±0.58 | 1.02±0.006 | 1.00±0.04 | 1.66±0.76 | 1.74±0.76 |
| Pei et al. ^[132]^ | T2DM patients | 60 | 7 | 4.99±0.92 | 5.23±1.08 | 3.02±0.82 | 3.18±0.89 | 1.10±0.32 | 1.24±0.29 | 1.96±1.14 | 2.40±1.17 |
| Arnaiz-Villena et al. ^[133]^ | General population | 257 | 65 | 5.58±0.40 | 5.53±1.49 | - | - | 1.20±0.36 | 1.22±0.35 | 2.56±1.84 | 2.78±1.75 |
| Liu et al. ^[34]^ | Hemodialysis patients | 89 | 10 | 4.4±0.6 | 4.5±0.6 | 2.6±0.6 | 2.5±0.5 | 0.98±0.3 | 0.96±0.3 | 1.9±1.4 | 2.1±1.4 |
| Baldani et al. ^[58]^ | PCOS patients | 106 | 45 | 4.6±0.4 | 4.5±0.3 | 2.7±0.4 | 2.6±0.4 | 1.3±0.1 | 1.4±0.1 | 1.4±0.4 | 1.3±0.3 |
| Gu et al. ^[35]^ | General population | 459 | 361 | 4.10±1.16 | 4.21±1.07 | 1.35±0.43 | 1.33±0.39 | 1.24±0.37 | 1.22±0.36 | 1.94±1.80 | 2.95±2.22 |
| Hsiao et al. ^[13]^ | Females | 251 | 32 | 5.09±0.96 | 4.67±0.87 | - | - | - | - | 1.19±0.8 | 1.05±0.49 |
|  | Males | 355 | 25 | 4.91±0.86 | 5.14±1.08 | - | - | - | - | 1.52±1.01 | 1.61±0.98 |
| Rocha et al. ^[135]^ | MetS patients | 138 | 16 | 4.75±1.02 | 4.87±1.23 | 2.78±0.91 | 3.06±1.15 | 1.10±0.61 | 1.05±0.26 | 2.08±1.02 | 1.73±0.69 |
| Jiang et al. ^[26]^ | CAD patients | 208 | 55 | 5.31±1.27 | 6.22±1.45 | 3.51±1.42 | 4.13±1.21 | 1.17±0.35 | 1.12±0.28 | 1.69±0.98 | 1.80±0.89 |
|  | Non-CAD control subjects | 135 | 72 | 4.64±1.13 | 4.73±1.22 | 2.35±0.74 | 2.44±0.52 | 1.45±0.41 | 1.38±0.42 | 1.72±0.80 | 1.70±0.81 |
| Stryjecki et al. ^[59]^ | General population | 1067 | 377 | 4.07±0.88 | 4.08±0.84 | 2.66±0.7 | 2.62±0.63 | 1.3±0.33 | 1.33±0.35 | 1.07±0.57 | 1.02±0.54 |
| Rotter et al. ^[136]^ | General population | 186 | 86 | 5.42±1.54 | 5.57±1.42 | 3.59±1.48 | 3.73±1.28 | 1.10±0.32 | 1.10±0.33 | 1.64±0.93 | 1.61±0.87 |
| Li et al. ^[137]^ | Cerebral infarction | 274 | 28 | 4.75±1.03 | 4.79±1.09 | 2.68±0.78 | 2.79±0.82 | 1.25±0.44 | 1.14±0.30 | 1.76±0.99 | 1.82±0.70 |
| Grygiel-Gorniak et al. ^[69]^ | General population | 185 | 86 | 5.97±1.09 | 5.96±0.99 | 3.73±0.99 | 3.66±0.85 | 1.65±0.37 | 1.66±0.40 | 1.30±0.57 | 1.38±0.69 |
| Aberle et al. ^[41]^ | Obesity patients | 361 | 101 | 6.52±1.61 | 6.25±1.7 | 4.29±1.58 | 4.11±1.58 | 1.31±0.4 | 1.18±0.36 | 2.48±2.03 | 2.41±0.73 |
| Hasan et al. ^[27]^ | CAD patients | 82 | 18 | 5.34±1.4 | 6.18±0.71 | 3.57±1.33 | 4.4±0.64 | 0.86±0.22 | 0.79±0.05 | 1.91±0.65 | 2.15±0.3 |
|  | T2DM patients | 92 | 13 | 5.33±1.18 | 5.55±0.4 | 3.14±0.96 | 3.37±0.32 | 1.31±0.37 | 1.3±0.12 | 1.95±1.12 | 1.92±0.35 |
|  | CAD/T2DM patients | 77 | 23 | 5.27±1.18 | 5.92±0.58 | 3.36±1.1 | 3.95±0.66 | 0.88±0.18 | 0.93±0.15 | 2.15±0.74 | 2.3±0.4 |
| Zaki et al. ^[18]^ | PCOS patients | 68 | 32 | 3.22±0.28 | 4.78±0.36 | 2.94±0.67 | 4.21±0.55 | - | - | 1.31±0.23 | 1.63±0.15 |
|  | Healthy control subjects | 96 | 24 | 3.10±0.27 | 3.04±0.31 | 2.94±0.67 | 2.91±0.55 | - | - | 1.31±0.13 | 1.29±0.12 |
| Becer et al. ^[36]^ | Obesity patients | 98 | 62 | 5.91±0.87 | 6.12±084 | 3.43±0.96 | 3.64±0.71 | 1.28±0.23 | 1.22±0.21 | 1.65±0.42 | 1.81±0.4 |
|  | Non-obesity control subjects | 81 | 59 | 5.14±0.78 | 5.43±0.72 | 3.02±0.58 | 3.09±0.65 | 1.50±0.20 | 1.46±0.21 | 1.13±0.36 | 1.19±0.35 |
| Zheng et al. ^[139]^ | T2DM patients | 133 | 17 | 4.73±0.96 | 5.04±1.1 | 2.97±0.86 | 3.18±0.75 | 1.17±0.27 | 1.21±0.24 | 1.75±0.88 | 1.96±1.10 |
| Rahimi et al. ^[37]^ | PCOS patients | 235 | 48 | 3.34±0.79 | 3.47±0.85 | 1.92±0.63 | 1.97±0.7 | 1.21±0.34 | 1.15±0.27 | 0.86±0.45 | 1.14±0.67 |
| Saeidi et al. ^[25]^ | Acne vulgaris patients | 155 | 35 | 3.47±0.79 | 3.36±0.85 | 2.02±0.67 | 1.87±0.68 | 1.29±0.33 | 1.22±0.29 | 0.94±0.56 | 0.99±0.69 |
|  | Control subjects | 137 | 46 | 3.19±0.71 | 3.76±0.81 | 1.78±0.51 | 2.12±0.69 | 1.07±0.26 | 1.12±0.29 | 0.85±0.36 | 1.35±0.56 |
| Almeida et al. ^[60]^ | Overweight/obesity patients | 64 | 12 | 4.41±0.93 | 4.40±0.56 | 2.66±0.74 | 2.66±0.41 | 1.36±0.27 | 1.35±0.17 | 0.85±0.33 | 0.69±0.24 |
|  | Normal weight subjects | 140 | 35 | 4.42±0.63 | 4.52±0.74 | 2.40±0.55 | 2.5±0.52 | 1.56±0.27 | 1.52±0.29 | 0.66±0.25 | 0.66±0.18 |
| Chmurzynska et al. ^[140]^ | Central obesity patients | 95 | 47 | 5.92±1.15 | 6.04±1.07 | 3.7±1.01 | 3.85±0.95 | 1.39±0.27 | 1.43±0.27 | 1.88±1.26 | 1.69±0.58 |
| García-Ricobaraza et al. ^[141]^ | Pregnant women | 118 | 19 | 6.36±1.29 | 6.55±1.14 | 3.47±1.06 | 3.44±0.89 | 1.78±0.47 | 1.87±0.34 | 2.52±0.91 | 2.69±1.62 |
| Szkup et al. ^[61]^ | General population | 294 | 131 | 5.64±1.1 | 5.68±1.59 | 3.64±1.68 | 3.59±1.57 | 1.64±0.44 | 1.59±0.47 | 1.34±0.74 | 1.31±0.71 |
| Carrillo-Venzor et al. ^[142]^ | General population | 290 | 122 | 3.67±0.62 | 3.57±0.6 | 2.02±0.49 | 1.97±0.44 | - | - | - | - |
| Vales-Villamarín et al. ^[29]^ | Males | 535 | 98 | 4.69±0.65 | 4.75±0.80 | 2.76±0.64 | 2.87±0.75 | 1.56±0.34 | 1.51±0.31 | 0.80±0.29 | 0.81±0.24 |
|  | Females | 523 | 98 | 4.77±0.74 | 4.66±0.54 | 2.87±0.69 | 2.78±0.69 | 1.53±0.35 | 1.49±0.29 | 0.83±0.30 | 0.84±0.24 |

*PPARG*, peroxisome proliferator-activated receptor gamma gene; TC, total cholesterol; LDL-C, low-density lipoprotein cholesterol; HDL-C, high-density lipoprotein cholesterol; TG, triglycerides; T2DM, type 2 diabetes mellitus; PCOS, polycystic ovarian syndrome; CAD, coronary artery disease; MI, myocardial infarction; ACS, acute coronary syndrome; NAFLD, nonalcoholic fatty liver disease; MetS, metabolic syndrome; OSAHS, obstructive sleep apnea-hypopnea syndrome; HIV, human immunodeficiency virus.

**Table S5.** Original data of the obesity indexes of the subjects with different genotypes of the rs3856806 polymorphism in *PPARG*.

| **Authors, reference** | **Subjects** | **n** | | **BMI, kg/m^2^** | | **WC, cm** | | **WHR** | |
| --- | --- | --- | --- | --- | --- | --- | --- | --- | --- |
|  |  | CC | CT+TT | CC | CT+TT | CC | CT+TT | CC | CT+TT |
| Meirhaeghe et al. ^[72]^ | Obesity patients | 82 | 34 | 34.0±3.2 | 34.0±3.6 | - | - | 0.92±0.09 | 0.92±0.09 |
|  | Non-obesity control subjects | 520 | 183 | 24.2±2.9 | 24.6±2.7 | - | - | 0.86±0.09 | 0.87±0.09 |
| Wang et al. ^[67]^ | CAD/control males | 339 | 145 | 28.0±3.68 | 28.1±3.57 | 100.3±12.89 | 101.7±13.25 | 0.97±0.18 | 0.98±0.12 |
|  | CAD/control females | 112 | 51 | 28.5±5.29 | 27.6±4.28 | 92.4±14.82 | 90.0±13.57 | 0.87±0.01 | 0.85±0.07 |
| Peng et al. ^[75]^ | CAD/healthy subjects | 209 | 98 | 23.61±2.78 | 23.71±2.59 | - | - | - | - |
| Song et al. ^[79]^ | Nephropathy patients | 153 | 72 | 22.6±3.0 | 22.4±2.6 | - | - | - | - |
| Arashiro et al. ^[80]^ | Obesity males | 47 | 10 | 22.8±0.3 | 23.6±0.9 | - | - | - | - |
|  | Obesity females | 37 | 11 | 22.9±0.3 | 22.3±0.5 | - | - | - | - |
| Chao et al. ^[86]^ | Heavy smokers | 13 | 14 | 23.7±2.5 | 23.56±3.00 | - | - | - | - |
| Maeda et al. ^[63]^ | T2DM patients | 101 | 39 | 21.4±2.6 | 22.5±3.8 | - | - | - | - |
| Tai et al. ^[6]^ | T2DM patients | 274 | 146 | 26.83±6.79 | 27.22±6.52 | - | - | - | - |
|  | IGT patients | 319 | 219 | 25.69±6.61 | 25.65±6.51 | - | - | - | - |
|  | Normal subjects | 1783 | 1297 | 23.44±5.91 | 23.82±6.12 | - | - | - | - |
| Tavares et al. ^[70]^ | T2DM patients | 167 | 40 | 30.7±7.4 | 30.0±5.5 | 92.0±8.9 | 93.6±7.9 | 0.93±0.12 | 0.95±0.14 |
|  | Healthy controls | 147 | 23 | 25.8±3.4 | 25.9±4.1 | 89.0±6.0 | 89.2±5.6 | 0.93±0.12 | 0.93±0.15 |
| Moon et al. ^[95]^ | Non-T2DM control subjects | 195 | 80 | 23.8±3.2 | 22.72±2.81 | - | - | 0.89±0.06 | 0.86±0.06 |
| Rhee et al. ^[98]^ | General population | 164 | 89 | 24.12±2.98 | 24.06±2.86 | 78.37±8.11 | 78.97±8.67 | - | - |
| Liu et al. ^[105]^ | General population | 496 | 296 | 25.08±3.56 | 24.98±3.72 | 85.81±11.92 | 85.14±12.21 | 0.85±0.07 | 0.85±0.07 |
| Hui et al. ^[107]^ | NAFLD patients | 53 | 43 | 25.3±2.2 | 24.9±1.6 | - | - | 0.9±0.1 | 0.9±0.1 |
|  | Non-NAFLD control subjects | 71 | 25 | 24.3±2.0 | 24.5±2.1 | - | - | 0.8±0.1 | 0.8±0.1 |
| Morini et al. ^[9]^ | Males | 183 | 28 | 25.0±3.5 | 27.7±5.4 | 89.8±10.6 | 93.1±14.9 | - | - |
|  | Females | 304 | 51 | 24.9±4.9 | 24.8±4.6 | 78.1±11.4 | 77.7±10.3 | - | - |
| Evangelisti et al. ^[109]^ | ACS/healthy subjects | 412 | 85 | 26.6±3.9 | 26.1±3.66 | - | - | - | - |
| Kotronen et al. ^[111]^ | General population | 472 | 147 | 26±2.73 | 25.98±3.21 | - | - | - | - |
| Haseeb et al. ^[71]^ | General population | 513 | 173 | 25.60±5.76 | 25.43±5.90 | 91.94±9.52 | 92.20±9.00 | 0.93±0.13 | 0.93±0.13 |
| Wan et al. ^[66]^ | CAD patients | 50 | 28 | 22.48±6.60 | 22.05±3.06 | - | - | - | - |
|  | T2DM patients | 55 | 31 | 24.69±4.65 | 23.62±3.71 | - | - | - | - |
|  | CAD/T2DM patients | 197 | 106 | 23.94±5.50 | 22.82±3.58 | - | - | - | - |
| Yilmaz-Aydogan et al. ^[40]^ | CAD/T2DM patients | 67 | 35 | 26.46±4.13 | 25.62±3.73 | - | - | - | - |
|  | CAD patients | 57 | 43 | 26.95±3.09 | 25.88±3.19 | - | - | - | - |
|  | Non-CAD control subjects | 52 | 53 | 25.36±3.55 | 25.56±3.67 | - | - | - | - |
| Chen et al. ^[127]^ | Schizophrenia males | 173 | 135 | 24.80±4.80 | 24.50±4.30 | 89.80±12.10 | 88.00±11.20 | - | - |
|  | Schizophrenia females | 167 | 124 | 25.10±4.80 | 26.30±4.90 | 85.40±13.00 | 88.40±12.60 | - | - |
| Zhou et al. ^[62]^ | CAD patients | 586 | 278 | 24.76±2.38 | 25.23±2.71 | - | - | - | - |
|  | Non-CAD control subjects | 595 | 413 | 23.42±2.46 | 24.05±2.73 | - | - | - | - |
| Bhatt et al. ^[33]^ | Obesity/NAFLD patients | 131 | 31 | 28.4±3.4 | 27.25±2.04 | 94.6±10.0 | 93.42±6.74 | - | - |
|  | Obesity patients | 153 | 20 | 27.0±3.1 | 26.36±3.47 | 90.5±9.3 | 86.34±8.10 | - | - |
| Liu et al. ^[34]^ | Hemodialysis patients | 64 | 35 | 21.6±3.6 | 21.4±3.5 | - | - | - | - |
| Chehaibi et al. ^[134]^ | Stroke/T2DM patients | 86 | 31 | 25.86±4.46 | 24.80±2.05 | - | - | - | - |
|  | Stroke patients | 57 | 22 | 26.26±3.44 | 26.02±3.15 | - | - | - | - |
| Chia et al. ^[68]^ | General population | 476 | 138 | 27.03±5.45 | 26.31±5.52 | 92.00±12.65 | 91.37±12.69 | 0.91±0.22 | 0.92±0.12 |
| Grygiel-Gorniak et al. ^[69]^ | General population | 194 | 77 | 29.67±5.86 | 28.83±7.41 | 90.79±13.10 | 89.33±17.09 | 0.84±0.07 | 0.83±0.09 |
| Song et al. ^[64]^ | CAD patients | 244 | 172 | 23.63±3.15 | 24.31±3.36 | - | - | - | - |
|  | Non-CAD control subjects | 138 | 75 | 24.22±3.31 | 24.20±3.33 | - | - | - | - |

BMI, body mass index; WC, waist circumference; WHR, waist-to-hip ratio; CAD, coronary artery disease; T2DM, type 2 diabetes mellitus; IGT, impaired glucose tolerance; ACS, acute coronary syndrome; NAFLD, nonalcoholic fatty liver disease.

**Table S6.** Original data of the serum lipid variables of the subjects with different genotypes of the rs3856806 polymorphism in *PPARG*.

| **Authors, reference** | **Subjects** | **n** | | **TC, mmol/L** | | **LDL-C, mmol/L** | | **HDL-C, mmol/L** | | **TG, mmol/L** | |
| --- | --- | --- | --- | --- | --- | --- | --- | --- | --- | --- | --- |
|  |  | CC | CT+TT | CC | CT+TT | CC | CT+TT | CC | CT+TT | CC | CT+TT |
| Meirhaeghe et al. ^[72]^ | Obesity patients | 82 | 34 | 5.80±1.07 | 5.92±1.09 | - | - | - | - | 1.70±1.14 | 1.57±0.97 |
|  | Non-obesity control subjects | 520 | 183 | 5.77±1.02 | 5.95±1.05 | - | - | - | - | 1.34±1.99 | 1.48±2.12 |
| Wang et al. ^[67]^ | CAD/control males | 339 | 145 | 5.4±0.92 | 5.3±0.83 | 3.84±1.1 | 3.37±0.83 | 0.99±0.18 | 1.01±0.24 | 2.1±1.1 | 2.0±0.83 |
|  | CAD/control females | 112 | 51 | 5.7±1.06 | 5.5±1.43 | 3.59±1.06 | 3.4±1.14 | 1.29±0.42 | 1.31±0.36 | 1.8±1.06 | 1.7±0.71 |
| Peng et al. ^[75]^ | CAD/healthy subjects | 209 | 98 | 4.61±0.96 | 4.42±1.03 | 2.78±0.85 | 2.59±0.89 | 1.2±0.31 | 1.16±0.29 | 1.55±0.91 | 1.54±0.88 |
| Arashiro et al. ^[80]^ | Obesity males | 47 | 10 | 4.71±0.71 | 4.91±0.98 | 2.79±0.71 | 3.08±0.98 | 1.53±0.35 | 1.55±0.57 | 0.84±0.54 | 0.67±0.32 |
|  | Obesity females | 37 | 11 | 4.73±0.94 | 4.50±0.69 | 2.97±0.79 | 2.74±0.60 | 1.40±0.31 | 1.42±0.26 | 0.95±0.27 | 0.73±0.22 |
| Chao et al. ^[86]^ | Heavy smokers | 13 | 14 | 4.63±0.57 | 4.98±1.13 | 2.94±0.63 | 3.15±1.00 | 1.10±0.25 | 1.14±0.34 | 1.60±1.75 | 1.41±0.75 |
| Maeda et al. ^[63]^ | T2DM patients | 101 | 39 | 4.39±0.86 | 4.64±1.29 | - | - | 1.33±0.50 | 1.09±1.43 | 1.34±0.68 | 1.87±1.39 |
| Tai et al. ^[6]^ | T2DM patients | 274 | 146 | 6.09±1.66 | 6.14±1.93 | 4.16±1.66 | 4.26±1.45 | 1.14±0.50 | 1.14±0.36 | 2.38±2.81 | 2.20±2.66 |
|  | IGT patients | 319 | 219 | 5.86±1.79 | 5.78±1.78 | 3.95±1.61 | 3.89±1.63 | 1.24±0.54 | 1.24±0.59 | 1.97±1.79 | 2.07±1.92 |
|  | Normal subjects | 1783 | 1297 | 5.42±1.69 | 5.47±1.44 | 3.48±1.27 | 3.50±1.44 | 1.32±0.42 | 1.32±0.72 | 1.43±1.27 | 1.42±1.28 |
| Tavares et al. ^[70]^ | T2DM patients | 167 | 40 | 4.90±1.39 | 4.78±0.80 | 3.4±1.3 | 3.2±0.8 | 1.1±0.3 | 1.2±0.3 | 2.05±0.99 | 1.78±0.97 |
|  | Healthy controls | 147 | 23 | 4.72±0.73 | 4.43±0.59 | 3.3±0.8 | 3.0±0.6 | 1.2±0.3 | 1.2±0.3 | 1.52±0.69 | 1.59±1.05 |
| Moon et al. ^[95]^ | Non-T2DM control subjects | 195 | 80 | - | - | - | - | 1.16±0.26 | 1.18±0.33 | 1.65±0.83 | 1.47±0.82 |
| Rhee et al. ^[98]^ | General population | 164 | 89 | 5.22±0.97 | 5.18±0.90 | 3.06±0.90 | 3.00±0.82 | 1.53±0.39 | 1.53±0.31 | 1.36±0.77 | 1.39±0.76 |
| Liu et al. ^[101]^ | CAD/ACS patients | 263 | 198 | 4.82±3.19 | 4.62±1.32 | 2.94±1.10 | 2.97±1.07 | 1.20±0.42 | 1.22±0.27 | 2.00±1.62 | 1.95±1.09 |
| Liu et al. ^[105]^ | General population | 496 | 296 | 5.14±1.06 | 5.15±1.20 | - | - | - | - | - | - |
| Hui et al. ^[107]^ | NAFLD patients | 53 | 43 | 5.8±2.4 | 5.6±1.0 | 3.6±0.9 | 3.6±0.7 | 2.0±0.8 | 1.9±0.9 | 3.8±0.9 | 3.1±0.7 |
|  | Non-NAFLD control subjects | 71 | 25 | 5.1±1.2 | 5.4±1.1 | 3.3±0.6 | 3.5±0.7 | 2.2±0.9 | 2.0±1.3 | 2.0±0.5 | 2.5±0.7 |
| Morini et al. ^[9]^ | Males | 183 | 28 | - | - | - | - | 1.21±0.32 | 1.15±0.28 | 1.24±0.77 | 1.36±0.77 |
|  | Females | 304 | 51 | - | - | - | - | 1.48±0.32 | 1.50±0.32 | 0.84±0.40 | 0.91±0.63 |
| Evangelisti et al. ^[109]^ | ACS/healthy subjects | 412 | 85 | 5.1±1.14 | 5.01±1.04 | 2.91±0.84 | 2.81±0.72 | 1.43±0.35 | 1.44±0.33 | 1.34±0.91 | 1.28±0.64 |
| Kotronen et al. ^[111]^ | General population | 472 | 147 | - | - | - | - | 1.22±0.27 | 1.26±0.31 | - | - |
| Haseeb et al. ^[71]^ | General population | 513 | 173 | 6.39±5.44 | 6.83±6.40 | - | - | 0.98±0.47 | 1.06±0.51 | 2.15±1.13 | 2.09±0.98 |
| Wan et al. ^[66]^ | CAD patients | 50 | 28 | 4.37±1.32 | 4.57±1.31 | 2.82±0.69 | 2.63±0.99 | 1.21±0.23 | 1.49±0.59 | 1.65±0.89 | 1.55±0.82 |
|  | T2DM patients | 55 | 31 | 4.56±0.89 | 4.36±0.67 | 2.77±0.76 | 2.55±0.64 | 1.10±0.37 | 1.25±0.39 | 1.93±1.74 | 1.70±0.92 |
|  | CAD/T2DM patients | 197 | 106 | 4.66±1.09 | 4.48±1.11 | 2.74±0.73 | 2.68±0.90 | 1.21±0.38 | 1.22±0.41 | 2.52±1.59 | 1.67±0.89 |
| Yilmaz-Aydogan et al. ^[40]^ | CAD/T2DM patients | 67 | 35 | 4.87±1.31 | 5.5±1.56 | 2.98± 1.01 | 3.37±1.23 | 0.95±0.25 | 1.03±0.18 | 1.61±0.71 | 2.34±1.78 |
|  | CAD patients | 57 | 43 | 5.63±1.66 | 5.51±1.17 | 3.59±1.27 | 3.60±1.03 | 1.04±0.19 | 1.05±0.19 | 1.58±0.78 | 1.44±0.6 |
|  | Non-CAD control subjects | 52 | 53 | 5.18±1.47 | 4.67±1.31 | 3.55±1.37 | 2.96±1.11 | 1.12±0.13 | 1.11±0.29 | 1.64±1.03 | 1.72±0.88 |
| Chen et al. ^[127]^ | Schizophrenia males | 173 | 135 | - | - | - | - | 1.20±0.34 | 1.13±0.34 | 1.71±1.39 | 1.87±1.54 |
|  | Schizophrenia females | 167 | 124 | - | - | - | - | 1.39±0.42 | 1.34±0.41 | 1.50±1.00 | 1.56±0.89 |
| Zhou et al. ^[62]^ | CAD patients | 586 | 278 | 4.23±1.13 | 4.07±1.08 | 2.67±1.18 | 2.52±0.78 | 1.07±0.35 | 1.16±0.62 | 1.83±1.26 | 1.67±0.75 |
|  | Non-CAD control subjects | 595 | 413 | 3.89±1.06 | 4.02±1.17 | 2.31±0.98 | 2.20±0.75 | 1.30±0.32 | 1.36±0.53 | 1.32±0.75 | 1.43±0.96 |
| Hung et al. ^[28]^ | HIV patients | 47 | 44 | 5.35±1.16 | 5.30±1.25 | 2.95±0.89 | 2.94±0.88 | 1.16±0.29 | 1.19±0.31 | 4.02±4.75 | 3.35±3.17 |
| Bhatt et al. ^[33]^ | Obesity/NAFLD patients | 131 | 31 | 4.91±0.84 | 4.74±0.62 | 2.87±0.36 | 2.87±0.34 | 1.02±0.16 | 0.99±0.13 | 1.94±0.84 | 1.92±0.82 |
|  | Obesity patients | 153 | 20 | 4.61±0.61 | 4.85±0.59 | 2.66±0.32 | 2.68±0.30 | 1.3±0.2 | 1.26±0.26 | 1.66±0.78 | 1.68±0.74 |
| Liu et al. ^[34]^ | Hemodialysis patients | 64 | 35 | 4.4±0.6 | 4.3±0.6 | 2.35±0.82 | 2.56±0.59 | 0.96±0.2 | 1.01±0.3 | 1.9±1.4 | 2.0±1.3 |
| Gu et al. ^[35]^ | General population | 418 | 402 | 4.03±1.14 | 4.27±1.09 | 1.34±0.43 | 1.35±0.39 | 1.22±0.39 | 1.24±0.33 | 2.03±1.77 | 2.75±2.27 |
| Chehaibi et al. ^[134]^ | Stroke/T2DM patients | 86 | 31 | 4.68±1.17 | 4.32±1.10 | 2.97±1.21 | 2.58±0.99 | 1.03±0.32 | 1.12±0.35 | 1.46±0.61 | 0.89±0.38 |
|  | Stroke patients | 57 | 22 | 4.58±1.16 | 4.41±1.17 | 2.80±1.23 | 2.65±1.14 | 1.13±0.45 | 1.05±0.39 | 1.44±0.58 | 1.19±0.52 |
| Chia et al. ^[68]^ | General population | 476 | 138 | 4.42±0.98 | 4.42±0.94 | 2.55±0.87 | 2.49±0.82 | 1.20±0.22 | 1.25±0.35 | 1.47±1.09 | 1.53±1.17 |
| Jiang et al. ^[26]^ | CAD patients | 183 | 80 | 5.03±1.38 | 4.96±1.29 | 3.81±1.03 | 3.74±0.87 | 1.21±0.33 | 1 .30±0.29 | 1.79±0.82 | 1.48±0.63 |
|  | Non-CAD control subjects | 129 | 78 | 4.62±1.04 | 4.69±1.17 | 2.44±0.71 | 2.37±0.55 | 1.46±0.38 | 1.58±0.45 | 1.77±0.83 | 1.58±0.79 |
| Wei et al. ^[65]^ | Stroke patients | 111 | 38 | 5.33±1.20 | 4.77±1.08 | 3.54±0.93 | 2.91±0.77 | 1.06±0.25 | 1.07±0.28 | 1.65±0.95 | 1.65±0.95 |
|  | Non-stroke control subjects | 79 | 46 | 4.86±1.20 | 4.37±0.79 | 3.07±0.98 | 2.59±0.67 | 1.04±0.26 | 1.10±0.26 | 1.42±0.80 | 1.24±0.52 |
| Grygiel-Gorniak et al. ^[69]^ | General population | 194 | 77 | 6.01±1.07 | 5.87±1.03 | 3.74±0.97 | 3.62±0.90 | 1.65±0.38 | 1.67±0.39 | 1.34±0.59 | 1.29±0.66 |
| Rahimi et al. ^[37]^ | PCOS patients | 186 | 97 | 3.38±0.79 | 3.33±0.82 | 1.95±0.63 | 1.9±0.66 | 1.21±0.34 | 1.17±0.3 | 0.92±0.52 | 0.88±0.48 |
| Song et al. ^[64]^ | CAD patients | 244 | 172 | 4.12±1.20 | 4.33±1.11 | 2.51±0.94 | 2.72±0.91 | 1.01±0.27 | 1.01±0.25 | 1.67±1.51 | 1.55±0.78 |
|  | Non-CAD control subjects | 138 | 75 | 4.08±0.98 | 3.98±0.92 | 2.42±0.73 | 2.36±0.64 | 1.11±0.30 | 1.06±0.28 | 1.41±0.77 | 1.49±0.93 |

*PPARG*, peroxisome proliferator-activated receptor gamma gene; TC, total cholesterol; LDL-C, low-density lipoprotein cholesterol; HDL-C, high-density lipoprotein cholesterol; TG, triglycerides; CAD, coronary artery disease; IGT, impaired glucose tolerance; T2DM, type 2 diabetes mellitus; ACS, acute coronary syndrome; NAFLD, nonalcoholic fatty liver disease; PCOS, polycystic ovarian syndrome; HIV, human immunodeficiency virus.

**Table S7.** The main contributors to the heterogeneity in the association analyses between the rs1801282 polymorphism in *PPARG* and obesity indexes as well as serum lipid variables.

| **Variables** | **Authors, reference** | **Subjects** | **Ethnicity** | **Gender** | **Age** |
| --- | --- | --- | --- | --- | --- |
| BMI | Hasan et al. ^[27]^ | CAD patients | African | M/F | Adults |
|  | Hasan et al. ^[27]^ | T2DM/CAD patients | African | M/F | Adults |
|  | Fan et al. ^[16]^ | CAD/healthy subjects | East Asian | M/F | Adults |
|  | Barbieri et al. ^[30]^ | General population | European Caucasian | M/F | Adults |
|  | Bhagat et al. ^[17]^ | Obesity patients | South Asian | M/F | Adults |
|  | Ben et al. ^[10]^ | Obesity patients | African | M | Adults |
|  | Li et al. ^[48]^ | T2DM patients | East Asian | M/F | Adults |
|  | Mirzaei et al. ^[113]^ | T2DM patients | West Asian | M/F | Adults |
|  | Mirzaei et al. ^[113]^ | Non-T2DM control subjects | West Asian | M/F | Adults |
|  | Morini et al. ^[9]^ | General population | European Caucasian | M | Adults |
|  | Yilmaz et al. ^[99]^ | Non-PCOS control subjects | West Asian | F | Adults |
|  | Danawati et al. ^[7]^ | Non-T2DM control subjects | South Asian | M/F | Adults |
| WC | Hasan et al. ^[27]^ | CAD patients | African | M/F | Adults |
|  | Zaki et al. ^[18]^ | PCOS patients | African | F | Adults |
|  | Zaki et al. ^[18]^ | Healthy control subjects | African | F | Adults |
|  | Bhagat et al. ^[17]^ | Obesity patients | South Asian | M/F | Adults |
|  | Li et al. ^[48]^ | T2DM patients | East Asian | M/F | Adults |
|  | Mirzaei et al. ^[113]^ | Non-obesity control subjects | West Asian | M/F | Adults |
|  | Yilmaz et al. ^[99]^ | Non-PCOS control subjects | West Asian | F | Adults |
| WHR | Szkup et al. ^[61]^ | General population | European Caucasian | F | Adults |
|  | Zaki et al. ^[18]^ | PCOS patients | African | F | Adults |
|  | Bhagat et al. ^[17]^ | Obesity patients | South Asian | M/F | Adults |
|  | Yang et al. ^[131]^ | PCOS patients | East Asian | F | Adults |
|  | Kim et al. ^[14]^ | General population | East Asian | F | Adults |
|  | Li et al. ^[48]^ | T2DM patients | East Asian | M/F | Adults |
| TC | Jiang et al. ^[26]^ | CAD patients | East Asian | M/F | Adults |
|  | Shen et al. ^[24]^ | CAD patients | East Asian | M/F | Adults |
|  | Chistiakov et al. ^[53]^ | Non-T2DM control subjects | European Caucasian | M/F | Adults |
|  | Zaki et al. ^[18]^ | PCOS patients | African | F | Adults |
|  | Saeidi et al. ^[25]^ | Non-acne vulgaris control subjects | West Asian | M/F | Adults |
|  | Mori et al. ^[74]^ | T2DM patients | East Asian | M/F | Adults |
| LDL-C | Jiang et al. ^[26]^ | CAD patients | East Asian | M/F | Adults |
|  | Regieli et al. ^[110]^ | CAD patients | European Caucasian | M | Adults |
|  | Shen et al. ^[24]^ | CAD patients | East Asian | M/F | Adults |
|  | Chistiakov et al. ^[53]^ | T2DM patients | European Caucasian | M/F | Adults |
|  | Zaki et al. ^[18]^ | PCOS patients | African | F | Adults |
|  | Saeidi et al. ^[25]^ | Non-acne vulgaris control subjects | West Asian | M/F | Adults |
| HDL-C | Yilmaz-Aydogan et al. ^[40]^ | Healthy subjects | West Asian | M/F | Adults |
|  | Shen et al. ^[24]^ | CAD patients | East Asian | M/F | Adults |
|  | Baldani et al. ^[58]^ | PCOS patients | European Caucasian | F | Adults |
|  | Chistiakov et al. ^[53]^ | Non-T2DM control subjects | European Caucasian | M/F | Adults |
|  | Montagnana et al. ^[51]^ | Hypertensive patients | European Caucasian | M/F | Adults |
|  | Chae et al. ^[52]^ | PCOS patients | East Asian | F | Adults |
|  | Swarbrick et al. ^[38]^ | Obesity patients | Australian Caucasian | M/F | Adults |
|  | Bhatt et al. ^[33]^ | Obesity/NAFLD patients | South Asian | M/F | Adults |
|  | Bhatt et al. ^[33]^ | Obesity subjects | South Asian | M/F | Adults |
|  | Aberle J et al. ^[41]^ | Obesity subjects | European Caucasian | M/F | Adults |
|  | Koika et al. ^[116]^ | PCOS patients | European Caucasian | F | Adults |
| TG | Youssef et al. ^[32]^ | Non-ACS control subjects | African | M/F | Adults |
|  | Lu et al. ^[49]^ | General population | East Asian | M/F | Adults |
|  | Shen et al. ^[24]^ | CAD patients | East Asian | M/F | Adults |
|  | Barbieri er al. ^[30]^ | General population | European Caucasian | M/F | Adults |
|  | Chistiakov et al. ^[53]^ | T2DM patients | European Caucasian | M/F | Adults |
|  | Chistiakov et al. ^[53]^ | Non-T2DM control subjects | European Caucasian | M/F | Adults |
|  | Zaki et al. ^[18]^ | PCOS patients | African | F | Adults |
|  | Bhagat et al. ^[17]^ | Obesity patients | South Asian | M/F | Adults |
|  | Rahimi et al. ^[37]^ | PCOS patients | West Asian | F | Adults |
|  | Gu et al. ^[35]^ | General population | East Asian | M/F | Adults |
|  | Saeidi et al. ^[25]^ | Non-acne vulgaris control subjects | West Asian | M/F | Adults |
|  | Becer et al. ^[36]^ | Obesity patients | West Asian | M/F | Adults |
|  | Yang et al. ^[131]^ | PCOS patients | East Asian | M/F | Adults |
|  | González et al. ^[42]^ | Obesity/control subjects | European Caucasian | F | Adults |
|  | Mirzaei et al. ^[113]^ | Non-obesity controls | West Asian | M/F | Adults |
|  | Swarbrick et al. ^[38]^ | Obesity patients | Australian Caucasian | M/F | Adults |
|  | Kotani et al. ^[103]^ | General population | East Asian | F | Adults |
|  | Danawati et al. ^[7]^ | T2DM patients | South Asian | M/F | Adults |

*PPARG*, peroxisome proliferator-activated receptor gamma gene; BMI, body mass index; WC, waist circumference; WHR, waist-to-hip ratio; TC, total cholesterol; LDL-C, low-density lipoprotein cholesterol; HDL-C, high-density lipoprotein cholesterol; TG: triglycerides; T2DM, type 2 diabetes mellitus; PCOS, polycystic ovarian syndrome; CAD, coronary artery disease; ACS, acute coronary syndrome; M, male; F, female.

**Table S8.** The main contributors to the heterogeneity in the association analyses between the rs3856806 polymorphism in *PPARG* and BMI as well as serum lipid variables.

| **Variables** | **Authors, reference** | **Subjects** | **Ethnicity** | **Gender** | **Age** |
| --- | --- | --- | --- | --- | --- |
| BMI | Zhou et al. ^[62]^ | Non-CAD control subjects | East Asian | M/F | Adults |
|  | Arashiro et al. ^[80]^ | Obesity patients | East Asian | M | Children/adolescents |
|  | Arashiro et al. ^[80]^ | Obesity patients | East Asian | F | Children/adolescents |
|  | Morini et al. ^[9]^ | General population | European Caucasian | M | Adults |
|  | Moon et al. ^[95]^ | Non-T2DM control subjects | South Asian | M/F | Adults |
| TC | Yilmaz-Aydogan et al. ^[40]^ | CAD/T2DM patients | West Asian | M/F | Adults |
|  | Wang et al. ^[67]^ | CAD/CAD-free subjects | Australian Caucasian | M/F | Adults |
|  | Wei et al. ^[65]^ | Stroke patients | East Asian | M/F | Adults |
|  | Wei et al. ^[65]^ | Non-stroke control subjects | East Asian | M/F | Adults |
|  | Gu et al. ^[35]^ | General population | East Asian | M/F | Adults |
|  | Meirhaeghe et al. ^[72]^ | Non-obesity control subjects | European Caucasian | M/F | Adults |
| LDL-C | Wang et al. ^[67]^ | CAD/CAD-free subjects | Australian Caucasian | M | Adults |
|  | Wei et al. ^[65]^ | Stroke patients | East Asian | M/F | Adults |
|  | Wei et al. ^[65]^ | Non-stroke control subjects | East Asian | M/F | Adults |
|  | Song et al. ^[64]^ | CAD patients | East Asian | M/F | Adults |
| TG | Wan et al. ^[66]^ | CAD/T2DM patients | East Asian | M/F | Adults |
|  | Yilmaz-Aydogan et al. ^[40]^ | CAD/T2DM patients | East Asian | M/F | Adults |
|  | Gu et al. ^[35]^ | General population | East Asian | M/F | Adults |
|  | Chehaibi et al. ^[134]^ | Stroke/T2DM patients | African | M/F | Adults |
|  | Hui et al. ^[107]^ | NAFLD patients | East Asian | M/F | Adults |
|  | Hui et al. ^[107]^ | Non-NAFLD control subjects | East Asian | M/F | Adults |
|  | Maeda et al. ^[63]^ | T2DM patients | East Asian | M/F | Adults |

*PPARG*, peroxisome proliferator-activated receptor gamma gene; BMI, body mass index; TC, total cholesterol; LDL-C, low-density lipoprotein cholesterol; TG: triglycerides; T2DM, type 2 diabetes mellitus; CAD, coronary artery disease; NAFLD, nonalcoholic fatty liver disease; M, male; F, female.
